# S100A9‐Targeted Cowpea Mosaic Virus as a Prophylactic and Therapeutic Immunotherapy against Metastatic Breast Cancer and Melanoma

**DOI:** 10.1002/advs.202101796

**Published:** 2021-09-14

**Authors:** Young Hun Chung, Jooneon Park, Hui Cai, Nicole F. Steinmetz

**Affiliations:** ^1^ Department of Bioengineering University of California La Jolla San Diego CA USA; ^2^ Department of Nanoengineering University of California La Jolla San Diego CA USA; ^3^ Department of Radiology University of California La Jolla San Diego CA USA; ^4^ Institute for Materials Discovery and Design University of California La Jolla San Diego CA USA; ^5^ Center for Nano‐ImmunoEngineering University of California La Jolla San Diego CA USA; ^6^ Moores Cancer Center University of California La Jolla San Diego CA USA

**Keywords:** breast cancer, immunotherapy, melanoma, metastasis, S100A9

## Abstract

Prognosis and treatment of metastatic cancer continues to be one of the most difficult and challenging areas of oncology. Treatment usually consists of chemotherapeutics, which may be ineffective due to drug resistance, adverse effects, and dose‐limiting toxicity. Therefore, novel approaches such as immunotherapy have been investigated to improve patient outcomes and minimize side effects. S100A9 is a calcium‐binding protein implicated in tumor metastasis, progression, and aggressiveness that modulates the tumor microenvironment into an immunosuppressive state. S100A9 is expressed in and secreted by immune cells in the pre‐metastatic niche, as well as, post‐tumor development, therefore making it a suitable targeted for prophylaxis and therapy. In previous work, it is demonstrated that cowpea mosaic virus (CPMV) acts as an adjuvant when administered intratumorally. Here, it is demonstrated that systemically administered, S100A9‐targeted CPMV homes to the lungs leading to recruitment of innate immune cells. This approach is efficacious both prophylactically and therapeutically against lung metastasis from melanoma and breast cancer. The current research will facilitate and accelerate the development of next‐generation targeted immunotherapies administered as prophylaxis, that is, after surgery of a primary breast tumor to prevent outgrowth of metastasis, as well as, therapy to treat established metastatic disease.

## Graphical abstract

Metastatic cancer continues to be one of the main challenges of oncology. It is demonstrated that cowpea mosaic virus (CPMV) is immunostimulatory when injected directly into tumors. The current work utilizes S100A9‐targeted CPMV as both an immunoprophylaxis and therapeutic approach against metastatic models of breast cancer and melanoma.

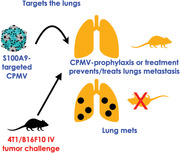

## 1 Introduction

Metastatic cancer remains a challenge to treat and diagnose regardless of the cancer's origin. For instance, the median survival time of breast cancer patients with metastatic recurrences is 2–3 years.^[^ ^1^ ^]^ Metastatic melanoma tumors are similar in their aggressiveness and prognosis of the disease becomes very difficult once metastasis has been achieved by the primary tumor.^[^ ^2^ ^]^ Metastasis to the lungs remains one of the most common forms of metastasis in both breast cancer and melanoma. In autopsy studies, lung metastasis in breast cancer was found in 57–77% of patients and found between 10% and 40% in melanoma patients.^[^ ^3^, ^4^ ^]^ Once lung metastasis occurs, the median survival rate of breast cancer patients is 22 months while in melanoma, overall survival is around 13 months.^[^ ^4^, ^5^ ^]^ Systemic chemotherapeutics are the primary treatment for metastatic disease but are limited by dose‐limiting toxicity, and suboptimal dosing can lead to drug resistance.^[^ ^6^ ^]^ Further, chemotherapy induces long‐term side effects—in melanoma, chemotherapy can lead to skin and gastrointestinal toxicity.^[^ ^7^ ^]^ Targeted therapies can help reduce off‐target effects.

A potential target in cancer therapy is S100A9. S100A9, otherwise known as myeloid‐related protein 14, is a central mediator of inflammation in cancer and other diseases.^[^ ^8^, ^9^ ^]^ It is a calcium‐binding protein that regulates inflammation and while there is some level of endogenous S100A9 expression in the squamous epithelium and mucosal tissues,^[^ ^9^, ^10^ ^]^ it becomes overexpressed in many different forms of cancer including breast, ovarian, skin, bladder, pancreatic, gastric, esophageal, colon, glioma, cervical, hepatocellular, and thyroid making it a potentially useful and ubiquitous target for therapeutics.^[^ ^8^, ^11^, ^12^, ^13^ ^]^ It is most commonly found in its heterodimer form with S100A8, but can also be found as a homodimer.^[^ ^12^, ^14^, ^15^ ^]^ S100A8/9 complexes are also found in mice and extensive biochemical characterization has demonstrated functional equivalency with its human counterpart.^[^ ^16^ ^]^ S100A9 expression is heavily linked with tumor aggressiveness and tumorigenesis through the activation of the nuclear factor‐*κ*B (NF‐*κ*B) and mitogen‐activated protein kinase pathways, which are responsible for inflammation‐induced cancer development and uncontrolled cell proliferation respectively.^[^ ^17^, ^18^ ^]^ It is mainly expressed and secreted by myeloid derived suppressor cells (MDSCs), which promotes further accumulation of MDSCs via autocrine pathways into the tumor microenvironment (TME) in an expanding and cyclic fashion.^[^ ^18^ ^]^ MDSCs suppress the immune response within the TME through reprogramming of the TME into a protumor phenotype, and tumors soon begin establishing S100A9 gradients of myeloid cell migration.^[^ ^19^, ^20^ ^]^ All the downstream effects of S100A9 establishment within the TME point to a clear link between S100A9 expression and tumor progression and metastasis.^[^ ^8^, ^11^, ^12^, ^13^ ^]^ Building on this knowledge, small molecule drugs and antibodies targeting S100A9 are being investigated as novel targeted therapeutics.^[^ ^21^, ^22^ ^]^ Kwak et al. generated peptides called H6 (MEWSLEKGYTIK) and G3 (WGWSLSHGYQVK) that were found through phage display and target S100A9.^[^ ^22^ ^]^ Kwak et al. fused these peptides to the Fc region of mouse IgG2b antibodies (termed peptibodies) and found that these peptibodies were successful in depleting MDSCs in multiple tumor models in the blood, spleen, and tumor leading to tumor growth inhibition.^[^ ^22^ ^]^ Similarly, neutralizing antibodies blocking S100A9 inhibited MDSC accumulation and decreased expression of serum amyloid 3, a recruiter of circulating tumor cells.^[^ ^21^ ^]^ Outside of these therapies, targeting S100A9 in cancer immunotherapy is a novel concept that has not been explored to the best of our knowledge.^[^ ^8^, ^22^ ^]^

Here, we sought to investigate the potential of an S100A9‐targeted immunotherapy using a plant virus nanotechnology. We previously demonstrated the potency of cowpea mosaic virus (CPMV) as an in situ vaccine technology, demonstrating that intratumoral administration of CPMV conferred efficacy against multiple tumor mouse models^[^ ^23^, ^24^, ^25^, ^26^ ^]^ and in canine cancer patients.^[^ ^27^ ^]^ In this previous work, CPMV was administered directly into an identified tumor; while non‐infectious toward mammals, the plant virus nanoparticles (VNPs) act as an adjuvant that activate the immune system via recognition of multiple pattern recognition receptors, namely toll‐like receptor (TLR)‐2, 4, and 7.^[^ ^28^ ^]^ The CPMV‐mediated local innate immune activation leads to remodeling of the TME and recruitment and activation of innate immune cells. This is followed by tumor cell killing (mediated by neutrophils and natural killer cells)^[^ ^26^, ^29^ ^]^ and tumor antigen processing, which ultimately activates the adaptive arm resulting in cell‐mediated systemic anti‐tumor immunity.^[^ ^26^, ^29^, ^30^ ^]^ The systemic immunity and immunological memory thus protects from recurrence, a key factor in fighting off metastatic cancers.^[^ ^26^ ^]^

We built on these prior studies, but set out to develop a CPMV immunotherapy for systemic administration targeting S100A9. Tumors establish a S100A9 gradient for myeloid cell migration.^[^ ^19^, ^20^ ^]^ Therefore, we hypothesized that we could utilize this gradient to direct S100A9‐targeted CPMV to sites of metastasis. We evaluated this approach in mouse models of lung metastasis from breast cancer (4T1‐luc in Balb/C) and melanoma (B16F10 in C57BL/6) and test efficacy of prophylactic and therapeutic approaches.

## 2 Results

### 2.1 S100A9‐Targeted Plant Virus Nanoparticles

**Figure 1:**
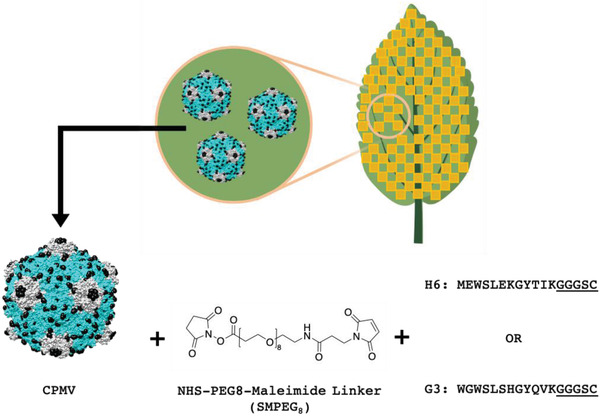
CPMV bioconjugation strategy. CPMV is first extracted from infected black‐eyed pea No. 5 plants. The large and small coat proteins are shown in blue and grey; surface exposed Lys side chains are highlighted as black spheres. The H6/G3 peptides with C‐terminal Cys side chain (the linker is underlined) were then conjugated to CPMV using an SMPEG_8_ linker via NHS‐maleimide chemistry. CPMV images and chemical structures were drawn with UCSF Chimera and ChemDraw software. The image of the leaf is created with BioRender.com.

**Figure 2:**
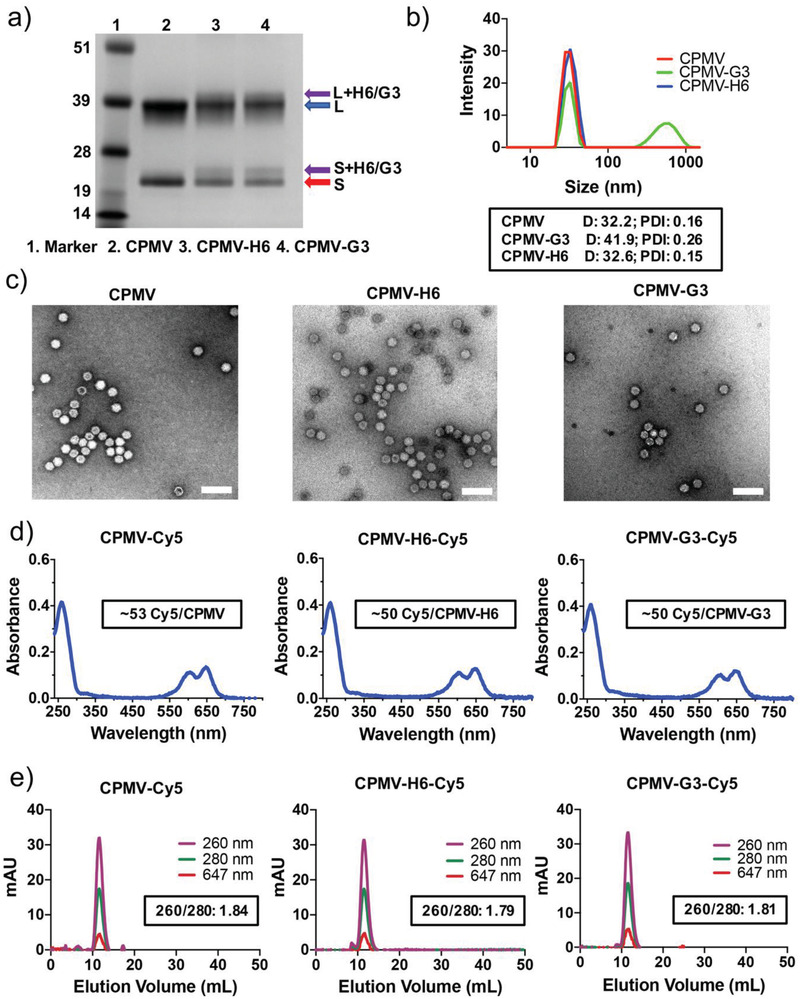
Characterization of CPMV, peptide‐conjugated CPMV, and fluorescent CPMV particles. a) SDS‐PAGE of the CPMV particles. The purple arrows point to H6/G3 peptide‐modified coat proteins. The blue arrow points to the large coat protein (42 kDa), and the red arrow points to the small coat protein (24 kDa). b) DLS measurements of the CPMV particles. The box in black is displaying the average diameter in nm of the particles (D) and the polydispersity index (PDI). c) TEM images of uranyl acetate‐stained CPMV particles. Scale bars represent 100 nm. d) UV–vis of the fluorescent Cy5‐conjugated CPMV particles. The boxed insets are displaying the number of conjugated Cy5 particles per CPMV particle (based on Beer's Law). e) FPLC measurements of the fluorescent and peptide‐conjugated CPMV particles. The inset is indicating the 260/280 nm ratio at the peak of the FPLC curve. Corresponding CCMV data are shown in Figure S2e, Supporting Information.

CPMV and cowpea chlorotic mottle virus (CCMV) control nanoparticles were purified from infected black‐eyed pea No. 5 plants. The CCMV nanoparticles served as a control because unlike CPMV, the CCMV nanoparticles do not elicit anti‐tumor immunity when used as in situ vaccine.^[^ ^31^ ^]^ H6 and G3 peptides were synthesized with a C‐terminal GGGSC linker for conjugation to the VNPs, which offer solvent‐exposed lysine side chains.^[^ ^32^, ^33^ ^]^ Conjugation was achieved via use of the heterobifunctional linker SMPEG_8_, where the *N*‐hydroxysuccinimide (NHS) ester reacts with lysines on CPMV/CCMV and the maleimide reacts with the cysteine on the peptide (**Figure** **1** and Figure S1, Supporting Information, respectively). To characterize peptide conjugation, the particles were denatured and the coat proteins (CPs) analyzed by sodium dodecyl sulfate‐polyacrylamide gel electrophoresis (SDS‐PAGE) (**Figure** **2**a and Figure S2a, Supporting Information). The capsid of CPMV particles consist of 60 copies each of a large and small CP (42 and 24 kDa, respectively) while CCMV particles consist of 180 copies of one 20 kDa CP. SDS‐PAGE confirmed successful conjugation with higher molecular weight bands detectable for the CPs. The molecular weight of the peptides are 1846 and 1809 g mol^−1^ for the H6 and G3 peptides, respectively; therefore, the band pattern is consistent with a mosaic of unmodified and peptide‐displaying CPs for both the CPMV and CCMV formulations (Figure 1, Figure S1, Supporting Information). Band analysis using ImageJ indicated roughly 20% of CPMV and 17% of CCMV CPs were conjugated to the peptide indicating ≈24 and 31 peptides per particle, respectively.

To validate the structural integrity of the S100A9‐targeted CPMV and CCMV formulations, native agarose gel electrophoresis, size exclusion chromatography (using fast protein liquid chromatography (FPLC)), and transmission electron microscopy (TEM) imaging was carried out. Native agarose gels on intact VNPs indicate stable capsids with minimal aggregation upon peptide conjugation (Figure S3a,b, Supporting Information). FPLC measurements of the S100A9‐targeted CPMV and CCMV particles show absence of any impurities such as free CP or broken particles with a single peak indicating monodisperse particles (Figure S3d,e, Supporting Information). Dynamic light scattering (DLS) measurements were consistent with the reported size of CPMV and CCMV^[^ ^26^, ^34^ ^]^ and indicate presence of monodisperse S100A9‐targeted nanoparticles with hydrodynamic diameters of approximately 30 nm (Figure 2b and Figure S2b, Supporting Information). The low polydispersity indices (Figure 2b and Figure S2b, Supporting Information, black box) indicate none to minimal aggregation of the particles after peptide conjugation. The G3‐conjugated CPMV and CCMV particles did showcase some level of aggregation although this was not deemed largely significant to warrant exclusion from future studies. Zeta potential measurements showed that the CPMV‐G3 and CPMV‐H6 formulation are less negatively charged compared to native CPMV (CPMV is −16.13 mV, CPMV‐H6 is −11.3 mV, and CPMV‐G3 is −9.78 mV) (Figure S3c, Supporting Information). CPMV‐G3 is less negative versus CPMV‐H6; while the differences are subtle, nanomaterials with surface charge close to neutral have a higher propensity to aggregate, and this may explain why CPMV‐G3 but not CPMV‐H6 formulations aggregate. Regardless, the TEM images show that both the CPMV and CCMV particles are structurally intact, measuring ≈30 nm, and with no morphological changes with or without peptide conjugation (Figure 2c and Figure S2c, Supporting Information).

For the biodistribution study, CPMV and CCMV nanoparticles were dual labeled with H6/G3 peptides and Cy5. S100A9‐targeted, fluorescent VNPs were analyzed by SDS‐PAGE and native agarose gel electrophoresis to confirm that the Cy5 label was covalently introduced—as evident by appearance of fluorescent protein bands (Figure S4a,b, Supporting Information). UV–vis measurements were used to determine the number of dyes per particle, and we found consistent labeling with ≈50 Cy5 labels conjugated to CPMV, CPMV‐H6, and CPMV‐G3 (Figure 2d). For CCMV, ≈35 Cy5 labels were conjugated to CCMV, CCMV‐H6, and CCMV‐G3 (Figure S2d, Supporting Information). Dye conjugation was quantified based on the UV–vis absorbance of Cy5 at 647 nm versus absorbance of CPMV at 260 nm using Beer's Law and the respective extinction coefficients for Cy5 and the VNPs. Fast protein liquid chromatograpahy (FPLC) analysis was consistent with intact and labeled VNPs being eluted from the column without any detectable free dye (Figure 2e and Figure S2e, Supporting Information). The dye co‐elutes (absorbance measured at 647) with the RNA and protein signals (measured as 260 and 280 nm, respectively) indicating successful conjugation.

### 2.2 Biodistribution of Native and S100A9‐Targeted Cowpea Mosaic Virus and Cowpea Chlorotic Mottle Virus

**Figure 3:**
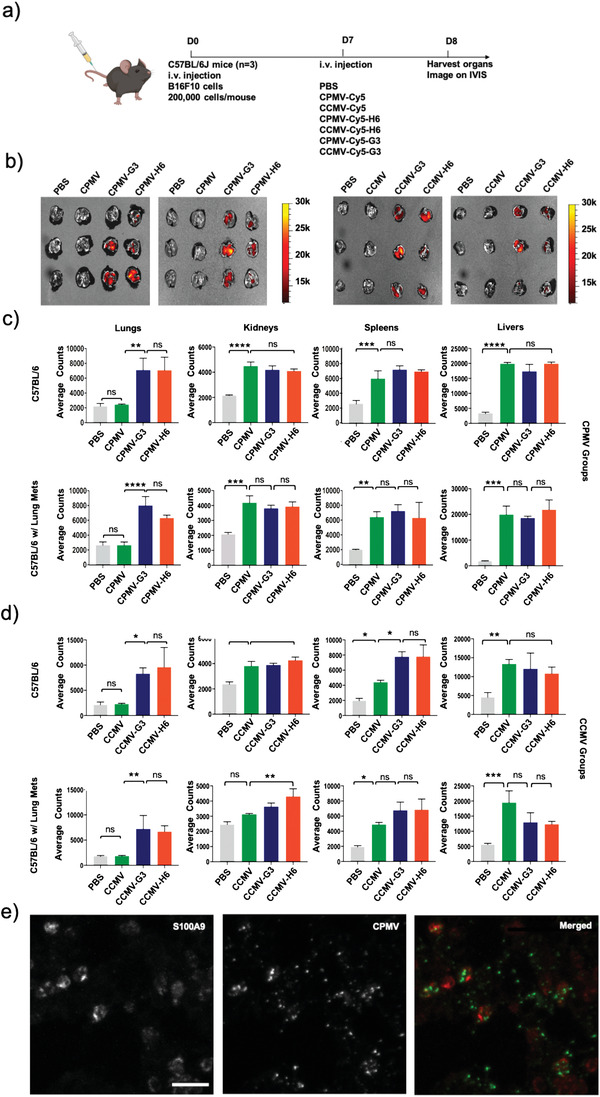
Biodistribution and localization of fluorescent CPMV and CCMV nanoparticles following administration. a) Schematic and timeline of the biodistribution study. b) IVIS imaging of lungs following CPMV and CCMV nanoparticle injection. Quantitative analysis of the fluorescence signal from the organs after c) CPMV and d) CCMV nanoparticle injection. e) Confocal imaging indicates co‐localization of the CPMV‐Cy5‐G3 particles with S100A9. Scale bar represents 5 µm. The merged image shows the S100A9 in red and the CPMV in green. All experiments contained a sample size of *n* = 3 and significance was deemed as *p* < 0.05. All analyses were performed by one‐way analysis of variance (ANOVA). * = *p* < 0.05, ** = *p*< 0.01, *** = *p* < 0.001, **** = *p* < 0.0001, ns = not significant. All instances of CPMV and CCMV in Figures (b–d) are fluorescent nanoparticles, but were not labeled as Cy5‐conjugated to improve image simplicity. The image of the mouse is created with BioRender.com.

To study biodistribution of VNPs, both Cy5‐labeled native and S100A9‐targeted CPMV and CCMV particles were intravenously (i.v.) injected in healthy and B16F10 metastatic tumor‐bearing C57BL/6J mice. After 24 h, mice organs (lung, liver, kidney, and spleen) were harvested and imaged (**Figure** **3**a, Figure S5b,c, Supporting Information). The fluorescent images show that CPMV and CCMV do not home to lungs and are mainly cleared by other organs such as the liver (75%), spleen (14–18%), and kidneys (6–10%) (Figure 3b,c and Figure S5b,c, Supporting Information, respectively), as previously reported.^[^ ^35^ ^]^ Likewise, the S100A9‐targetd CPMV and CCMV nanoparticles accumulated in the liver and spleen, but there was also significant accumulation within the lungs regardless of tumor inoculation. ≈19% of the CPMV‐Cy5‐G3 and 17% of the CPMV‐Cy5‐H6 nanoparticles accumulated within the lungs of healthy mice. Lung accumulation within tumor‐inoculated mice was similar with 18% and 12% accumulation of the CPMV‐Cy5‐G3 and CPMV‐Cy5‐H6, respectively. The lung homing was also reflected by reduced liver clearance, 55–68% for the S100A9‐targeted versus 75% for native CPMV. Differences in spleen or kidney deposition of targeted versus non‐targeted particles were not detected. Overall, the trend was similar for CCMV formulations. While native CCMV does not home to the lungs, H6/G3 conjugation led to significant CCMV lung homing of up to 35% in healthy mice and 29% in tumor‐bearing mice (Figure 3b,d). Homing to the healthy lung may be explained by S100A9 expression in mucosal tissues.^[^ ^9^, ^10^ ^]^

To investigate the co‐localization of the CPMV particles with S100A9, confocal imaging of tumor‐bearing lungs sections was performed. As evidenced from the ex vivo IVIS imaging (Figure 3b), Cy5‐labeled CPMV particles showed minimal to no accumulation in the lungs (not shown). In contrast, CPMV‐Cy5‐G3 particles strongly co‐localized with S100A9 expression in the lungs (Figure 3e). ImageJ co‐localization analysis using the Fiji Coloc2 platform reveals a Mander's M2 colocalization coefficient of 0.504 for CPMV:S100A9 indicating that indeed there is association of the CPMV‐Cy5‐G3 and S100A9. Ex vivo targeting data using splenocytes and tumor cells collected from mice bearing 4T1 subcutaneous (s.c.) tumors indicate that H6/G3 peptide‐conjugated CPMV target MDSCs with CPMV‐G3 showing higher binding versus CPMV‐H6, in particular with the polymorphonuclear‐MDSCs (Figure S6, Supporting Information). Native CPMV showed negligible binding which is consistent with previous data that indicate that CPMV in lung tumors is mostly taken up by neutrophils and to a lesser degree by macrophages and MDSCs.^[^ ^26^ ^]^ The targeting of MDSCs of CPMV‐H6/G3 is attributed to the peptides which have been shown to target other biologics to MDSCs.^[^ ^22^ ^]^

### 2.3 B16F10 i.v. Challenge to Cowpea Mosaic Virus and Cowpea Chlorotic Mottle Virus Pre‐Exposed Mice (Prophylaxis)

**Figure 4:**
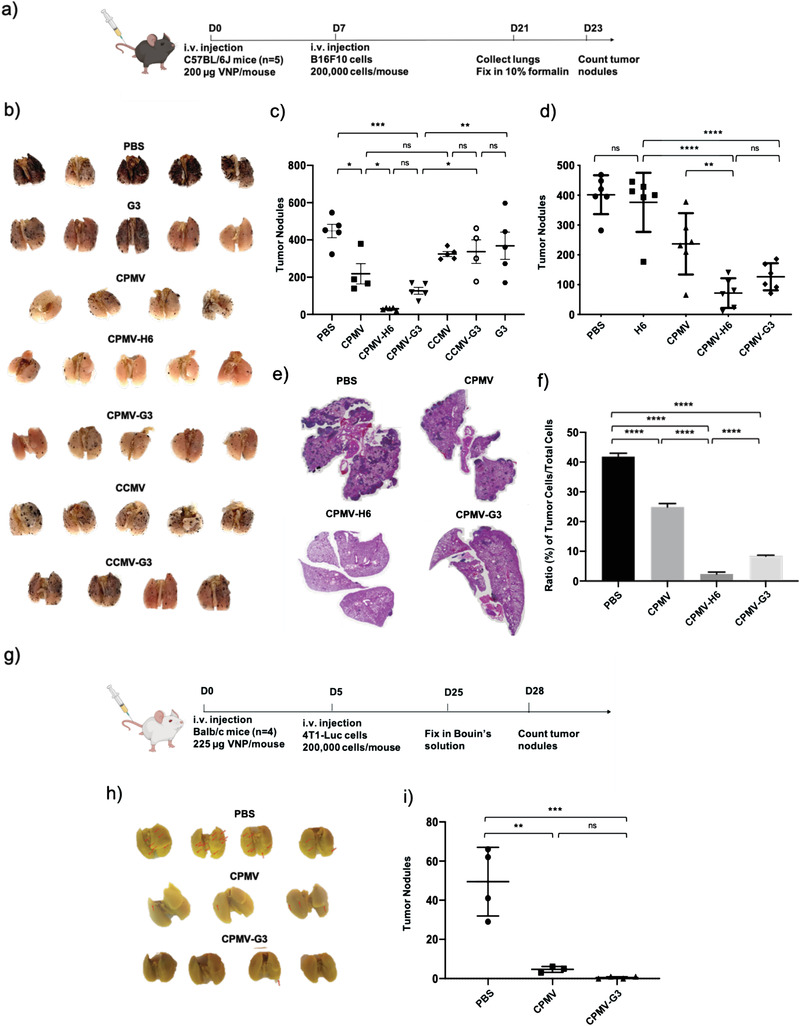
CPMV particles show immunoprophylaxis in C57BL/6J mice challenged i.v. with B16F10 melanoma or 4T1‐Luc breast cancer cells. a) Schematic and timeline of the B16F10 prophylaxis study. b) Harvested lungs were fixed and imaged before manual tumor counting. c) Quantitative analysis of the number of tumor nodules found on the surface of the lungs. The + sign indicates the mean while the solid horizontal line indicates the median. d) Repeated B16F10 prophylactic immunotherapy study including an H6 peptide only control. The middle line indicates the mean number of tumor nodules. e) H&E images of the harvested lungs. The dark purple spots are indicative of the B16F10 tumor nodules in the lungs. f) Quantitative analysis of the H&E pictures in e). The ratio of tumor cells to total cells within the H&E images were plotted. The images were analyzed using QuPath software. g) Schematic and timeline of the 4T1‐Luc prophylaxis study. h) Harvested lungs were fixed in Bouin's solution before manual tumor counting. The tumor nodules are highlighted by the red arrows. i) Quantitative analysis of the tumor nodules from the lungs in h). All B16F10 experiments had a sample size of *n* = 4–5 while 4T1‐Luc experiments were accomplished with a sample size of *n* = 4. All analyses were done by one‐way ANOVA, and significance was deemed as *p* < 0.05.* = *p* < 0.05, ** = *p* < 0.01, *** = *p* < 0.001, **** = *p* < 0.0001, ns = not significant. The image of the mouse is created with BioRender.com.

To investigate the suitability of S100A9‐targeted VNPs to serve as a prophylactic immunotherapy preventing manifestation of lung metastasis, we used a lung metastasis mouse model using C57BL/6J mice i.v. challenged with B16F10 melanoma cells. C57BL/6J mice were pre‐exposed to CPMV and CCMV nanoparticles with and without the H6/G3 targeting ligands one week before i.v. B16F10 challenge (**Figure** **4**a). Lungs were harvested 2 weeks post tumor challenge for tumor nodule counting and histology. Native CPMV treatment resulted in a 2.1‐fold (*p* = 0.0168) decrease in formation of tumor nodules compared to the phosphate buffered saline (PBS) control while lungs harvested from mice treated with CPMV‐H6 and CPMV‐G3 nanoparticles significantly decreased tumor burden in the lungs by 14.8 (*p* < 0.0001) and 3.5‐fold (*p* = 0.0002) compared to the PBS control, respectively (Figure 4b,c). The mean number of tumor nodules were 218 for CPMV, 30.2 for CPMV‐H6, 126.6 for CPMV‐G3, and 447.6 for PBS‐treated control animals (Figure 4c). Tumor burden in mice treated with the CCMV formulations and the G3 peptide only control was comparable to PBS treated mice. This experiment was repeated one more time with only the CPMV particles, as well as, the H6 peptide only control (Figure 4d). The repeated experiment produced very similar results; the mean number of tumor nodules were 237 for CPMV, 71.7 for CPMV‐H6, 126.3 for CPMV‐G3, and 401.5 for PBS‐treated control animals (Figure 4d). CPMV‐H6 reduced tumor nodules by 5.6 (*p* < 0.0001) and 3.2‐fold (*p* < 0.0001) compared to PBS and H6, respectively. Native CPMV again showed some level of effectiveness (1.7‐fold reduction (*p* = 0.0081) compared to PBS) although it was to a lesser degree than S100A9‐targeted CPMV. The CPMV‐H6 formulations had a fivefold enhanced efficacy versus CPMV (*p* = 0.077) and CPMV‐G3 exhibited twofold increase in efficacy as compared to CPMV although this was deemed insignificant (*p* = 0.1212).

The lungs were further examined through histology and hematoxylin & eosin (H&E) staining (Figure 4e). Qualitatively, the histology slides exemplify that the CPMV treatment greatly reduces tumor burden. There is a stark decrease in tumor cells (dark purple) indicative of the B16F10 tumor nodules found in the lungs when injected with CPMV‐H6 and CPMV‐G3 compared to lungs treated with PBS and CPMV. The ratio of tumor cells to total cells in the lung sections, analyzed using QuPath software, corroborate the findings. CPMV‐H6 and CPMV‐G3 reduced the ratio by 18‐fold (*p* < 0.0001) and fivefold (*p* < 0.0001), respectively, compared to PBS. Again, native CPMV displayed efficacy yet at significantly lower levels achieving only 1.7‐fold reduction (*p* < 0.0001) (Figure 4f).

### 2.4 4T1‐Luc i.v. Challenge to Cowpea Mosaic Virus Pre‐Exposed Mice (Prophylaxis)

To investigate the prophylactic effect of S100A9‐targeted CPMV in a murine triple negative breast cancer (TNBC) model, we first exposed Balb/C mice to CPMV or S100A9‐targeted CPMV via i.v. injection, and then challenged mice with luciferase‐labeled 4T1 (4T1‐Luc) cells. This experimental lung metastatic model mimics metastatic TNBC. Tumor cell challenge was carried out 5 days post CPMV exposure (Figure 4g). The disease progression of 4T1 tumors in the lung was imaged using the in vivo bioluminescence imaging system (IVIS). Balb/C mice pre‐exposed with PBS showed that lung metastases established within 2 weeks post tumor cell challenge. By day 21, all the mice in the PBS group had to be sacrificed due to illness and significant weight loss (Figure S7b, Supporting Information). On the contrary, both CPMV and CPMV‐G3 pre‐exposed mice showed no signs of tumor growth by day 21. The weight of the mice in both CPMV groups was consistent throughout the experiment without any significant loss; no apparent side effects were observed (Figure S7b, Supporting Information). The lungs from the CPMV and CPMV‐G3 groups were harvested after 25 days and fixed in Bouin's solution before manual counting of tumor nodules (Figure 4h,i). Compared to PBS, the CPMV showed a 10.6‐fold decrease in tumor nodules (*p* = 0.0015) while the CPMV‐G3 demonstrated a 99‐fold decrease (*p* = 0.0005) (Figure 4h,i). There was no significant difference between the CPMV and the CPMV‐G3.

### 2.5 Investigating S100A9‐Targeted Cowpea Mosaic Virus as an Immunotherapy after Establishment of Tumors from B16F10 and 4T1‐Luc (Immunotherapy)

**Figure 5:**
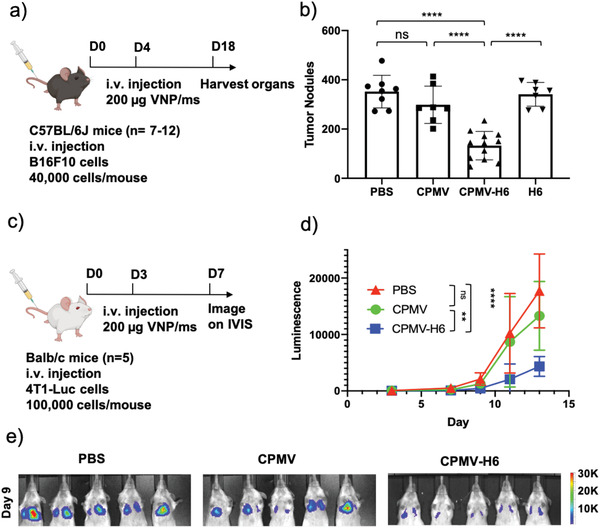
S100A9‐targeted CPMV immunotherapy against lung metastasis from i.v. injected B16F10 melanoma and 4T1‐Luc breast cancer cells in mice. a) Treatment schedule of the metastatic B16F10 melanoma model using C57BL/6J mice and therapeutic administration of CPMV and CPMV‐H6. b) Quantitative analysis of tumor nodules counted in lungs harvested post‐treatment. c) Treatment schedule of the metastatic 4T1‐Luc breast cancer model using Balb/c mice. d) Quantitative luminescence of the tumors following region of interest (ROI) measurements of the images from (e). e) Luminescent imaging of the 4T1‐Luc tumors taken on the IVIS. The mice were imaged every two days following 150 mg kg^−1^ i.p. injection of D‐luciferin, and the luminescence was calculated using ROI measurements from the Living Image 3.0 software. One representative image taken on the IVIS on day 9 is shown. For B16F10 experiments an *n* = 7–12 animals per group and for the 4T1‐Luc experiments an *n* = 5 animals per group were assigned. Statistical significance was characterized as *p* < 0.05. All analyses were done by either one or two‐way ANOVA. * = *p* < 0.05, ** = *p* < 0.01, *** = *p* < 0.001, **** = *p* < 0.0001, ns = not significant. The image of the mouse is created with BioRender.com.

S100A9‐targeted CPMV particles were tested as a potential immunotherapy against B16F10 and 4T1‐Luc mice tumor models. In this study, mice were first inoculated with B16F10 or 4T1‐Luc tumor cells, followed by the treatment with S100A9‐targeted CPMV (**Figure** **5**a,c). C57BL/6J mice (*n* = 7–12) were inoculated with B16F10 cells and treated once with PBS, CPMV, CPMV‐H6, or H6 peptide after 4 days (Figure 5a). After 18 days, lungs were harvested for tumor nodule counting. CPMV‐H6 particles as an immunotherapy demonstrated significant advantages compared to all the controls (Figure 5b). CPMV‐H6 treatment decreased tumor nodules by 2.7‐fold compared to PBS (*p* < 0.0001), 2.3‐fold compared to unconjugated CPMV (*p* < 0.0001), and 2.6‐fold compared to H6 peptide only (*p* < 0.0001). It is important to note that, unlike in the immunoprophylaxis, native CPMV (i.e., non‐targeted) did not show any significant decrease in tumor nodules compared to the PBS control.

The CPMV‐H6 particles were further tested in a 4T1‐Luc model by injecting female Balb/c mice (*n* = 5) with the particles 3 days post tumor inoculation (Figure 5c). Tumors in mice treated with PBS and native CPMV developed significantly by day 9 (Figure 5d,e). A representative bioluminescence image on day 9 is shown in Figure 5e while further imaging can be found in Figure S8, Supporting Information. Similar to the prophylactic immunotherapy study, the tumor burden in the lung was less severe and disease progression was delayed in the mice treated with CPMV‐H6 (Figure S8c, Supporting Information). The unabated tumor growth led to the mice in the PBS and CPMV groups all dying within 15 days or reaching their clinical endpoints before being sacrificed (Figure S8b, Supporting Information). The CPMV‐H6 treatment was able to extend the median time of survival by 3 days while the CPMV treatment extended the median time of survival by 1 day.

### 2.6 Immunogenicity of Cowpea Mosaic Virus and Cowpea Chlorotic Mottle Virus Nanoparticles

**Figure 6:**
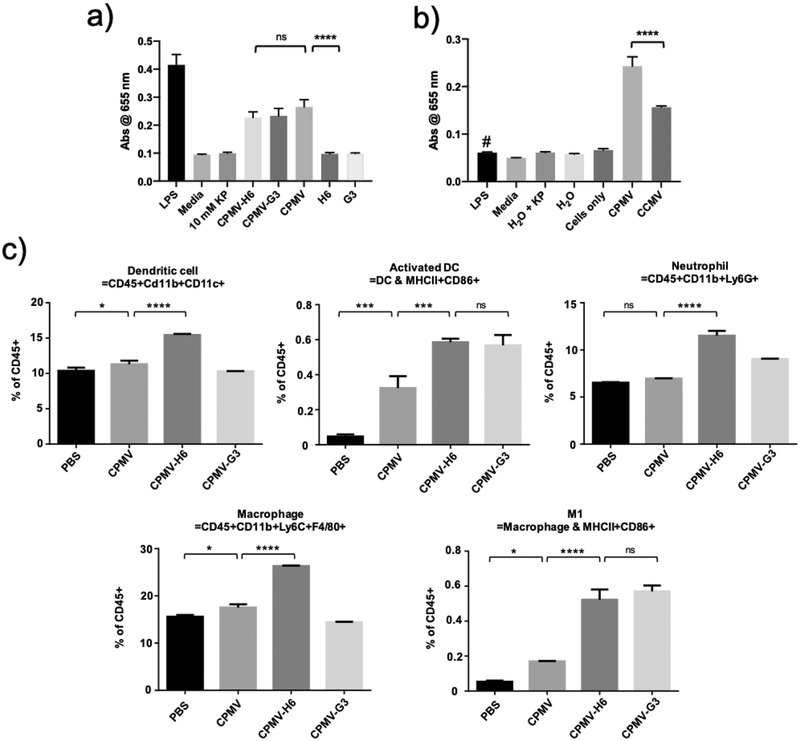
Immunogenicity assays of CPMV and CCMV particles. a) A RAW‐BLUE assay comparing the immunogenicity between wild type CPMV, peptide‐conjugated CPMV, and the peptide only controls; lipopolysaccharide (LPS) (10 µg was used as positive control). b) RAW‐BLUE assay comparing the immunogenicity of CPMV to CCMV. # Here LPS at ≈1 EU mL^−1^ served as control matched to the LPS contaminants found in the CPMV preparation. c) FACS analysis of the immune cell profile following CPMV injection. C57BL/6J mice were i.v. injected with CPMV, CPMV‐H6, CPMV‐G3, and PBS and the lungs were harvested and analyzed. FACS data was acquired with *n* = 3 animals per group, and significance was deemed as *p* < 0.05. All analyses were done using one‐way ANOVA. * = *p* < 0.05, ** = *p* < 0.01, *** = *p* < 0.001, **** = *p* < 0.0001, ns = not significant.

To gain insights into the underlying mechanism, we first evaluated the immunogenicity of targeted and native CPMV versus CCMV using a RAW‐BLUE assay (**Figure** **6**a,b). Following 24 h incubation with the particles and peptides, the wild type CPMV and the peptide‐conjugated CPMV exhibited higher level of activation of transcriptional factors (i.e., NF‐kB and AP‐1) compared to CCMV and negative controls. As expected, H6 and G3 peptides alone were not immunostimulatory indicating the peptides are not TLR and nucleotide‐binding oligomerization domain (NOD) agonists.

To assay whether CPMV targeting to the lungs would alter the immune cell profiles, lungs were collected 24 h post CPMV treatment and innate immune cell profiles were analyzed using fluorescence‐activated cell sorting (FACS). Indeed, data highlight considerable changes in the immune cell profiles in the lungs 24 h after particle administration (Figure 6c). While PBS and CPMV did not produce a significant impact (which is explained by the lack of lung accumulation, see Figure 3), CPMV‐H6 treatment in particular led to increased infiltration of leukocytes, especially dendritic cells (DC) and neutrophils. Compared to PBS, CPMV‐H6 increased the percentage of DCs in the lungs by 1.5‐fold (*p* < 0.0001) and neutrophils by 1.8‐fold (*p* < 0.0001). CPMV‐H6 also improved DC and neutrophil infiltration compared to native CPMV by 1.4‐fold (*p* < 0.0001) and 1.7‐fold (*p* < 0.0001), respectively. CPMV‐G3 did not significantly improve DC recruitment although it did increase neutrophil infiltration by 1.4 (*p* < 0.0001) and 1.3‐fold (*p* < 0.0001) compared to PBS and native CPMV, respectively. CPMV‐H6 additionally increased macrophage infiltration by 1.7 (*p* < 0.0001) and 1.5‐fold (*p* < 0.0001) compared to PBS and CPMV, respectively, although this effect was not observed with CPMV‐G3. When observing immune cell activation, CPMV‐G3 and CPMV‐H6 performed equally with insignificant differences between the two for DC activation and M1 macrophage polarization. However, against the controls, there was a significant improvement of DC activation by CPMV‐H6 and CPMV‐G3 with 12.5 (*p* < 0.0001) and 12.1‐fold (*p* = 0.0006) increases compared to PBS. Both CPMV‐H6/G3 increased DC activation by 1.8‐fold (*p* = 0.0006 for H6 and *p* = 0.0009 for G3). When comparing M1 activation, CPMV‐H6 improved activation by 9.9 (*p* < 0.0001) and 3.1‐fold (*p* < 0.0001) compared to PBS and CPMV, respectively while CPMV‐G3 similarly improved activation by 10.8 (*p* < 0.0001) and 3.4‐fold (*p* < 0.0001), respectively. CPMV was unable to generate as strong an immune cell response compared to the H6/G3 conjugated CPMV nanoparticles in all tested immune cell categories; this was expected because native CPMV did not accumulate in the lungs. The gating strategy used for the flow experiments can be found in Figure S9, Supporting Information.

## 3 Discussion

Targeted immunotherapies, such as, the S100A9‐targeted CPMV, could be a powerful treatment paradigm to treat high‐risk patients and prevent metastatic outgrowth. The standard of care for metastatic cancer is chemotherapy, but this often fails due to development of resistance and/or necessary dose reduction due to harsh side effects.^[^ ^36^, ^37^ ^]^ Alternatively, cancer immunotherapies have demonstrated that immune system modulation can result in dramatic antitumor activity. However, despite the enthusiasm surrounding clinical results using checkpoint inhibitor therapies,^[^ ^38^, ^39^ ^]^ there continues to be a need to develop approaches that take advantage of neoantigens^[^ ^40^, ^41^ ^]^ while overcoming therapy resistance.^[^ ^42^ ^]^ Immunotherapies reversing the immunosuppressive TMEs can mitigate some of these challenges and can be used as solo or combination therapies to launch systemic anti‐tumor immunity. We have previously demonstrated that CPMV as an in situ vaccine can act in this way, re‐polarizing immunosuppressed environments and promoting immune cell recruitment and activation.^[^ ^29^, ^43^ ^]^ In past studies, CPMV was administered intratumorally in mouse models of ovarian, breast, colon cancer, and melanoma.^[^ ^23^, ^26^, ^30^, ^45^, ^46^ ^]^ The CPMV nanoparticles are recognized by innate immune cells and signal through pattern recognition receptors leading to release of immunostimulatory cytokines including interleukin (IL)‐1*β*, IL‐12, interferon (IFN)‐*γ*, chemokine ligand 3, macrophage inflammatory protein‐2, and granulocyte‐macrophage colony‐stimulating factor leading to monocyte recruitment.^[^ ^23^, ^26^ ^]^ Activated DCs travel to nearby lymph nodes activating both CD4+ and CD8+ T‐cells to establish immune memory. Therefore, direct intratumoral injection of CPMV is an effective strategy to induce systemic anti‐tumor immunity, but is limited to injectable tumors. Here, we expand upon this concept and demonstrate that multivalent display of S100A9‐targeting ligands directs the CPMV nanoparticles to the lung TME and induces treatment as evident by reduced tumor burden in the lungs after mice were i.v. challenged using melanoma cells or TNBCs (Figure 4). We demonstrate that i.v. administered, S100A9‐targeted CPMV homes to the lungs and that the CPMV nanoparticle adjuvant effectively immunomodulates the lung environment to recruit DCs and neutrophils while polarizing macrophages to the M1 phenotype protecting mice from i.v. challenge with melanoma and TNBC. The S100A9‐targeted CPMV also was effective in treating lung metastasis from melanoma or TNBC after establishment of the disease.

We used S100A9 to deliver CPMV to lungs because lung metastases are common in various cancers and its prognosis is poor.^[^ ^47^ ^]^ In both men and women, lungs were the third highest site of metastasis while in specific cancers such as genital cancers, metastatic growth to the lungs was the most common. Once metastasis occurs, survival rates are low and novel therapies to extend survival must be continuously researched and implemented.^[^ ^4^, ^5^ ^]^ However, the concepts of targeting metastases in distant tissues could be expanded beyond just the lung. Many types of cancers including ovarian, skin, bladder, pancreatic, gastric, esophageal, colon, glioma, cervical, hepatocellular, and thyroid express S100A9.^[^ ^8^, ^11^, ^12^, ^13^ ^]^ S100A9 is also expressed in a wide range of cell types including granulocytes, monocytes, osteoclasts, early myeloid lineage cells, platelets, and cancer cells.^[^ ^13^, ^48^ ^]^ It can be expressed, secreted, or displayed, and secretion can be active or passive (i.e., neutrophil necrosis).^[^ ^17^, ^48^ ^]^ The fact that S100A9 is secreted and found throughout the TME makes it an attractive target to direct nanoparticles and immunotherapies to the disease site (Figure 3e). These design concepts could be applied to target other molecular signatures to tailor the nanoparticle treatment for organ‐specific metastatic niches.

The CPMV platform technology is a versatile technology that could be adapted to target other disease biomarkers and/or deliver additional payloads.^[^ ^49^, ^50^ ^]^ Here, we developed CPMV displaying peptide ligands specific for S100A9. Characterization of the S100A9‐targeted nanoparticles of CPMV (as well as, the CCMV control particles) demonstrated stable formulation chemistry, as demonstrated by DLS, FPLC, and TEM which indicate the lack of substantial aggregation and structural uniformity of the viruses regardless of conjugation (Figure 2 and Figure S2, Supporting Information). Denatured gels indicate a mosaic of conjugated and unconjugated coat proteins with up to 24 and 31 peptides per CPMV and CCMV nanoparticle, respectively. This equates to roughly 20% and 17% coat protein conjugation. Given the fairly low molecular weight of the SMPEG_8_ linker (MW: 689.71 g mol^−1^), the PEG chain is not expected to reduce the immunogenicity of the S100A9‐targeted CPMV. Also, previous studies have shown that PEGylation does not affect in situ efficacy of CPMV.^[^ ^45^ ^]^ Overall, the facile conjugation scheme producing monodisperse and highly conjugated viral nanoparticles is a key determinant in advancing the translatability and scalability of the CPMV platform. The LPS concentration in the CPMV preparation was 0.63 EU per mg protein; therefore, the 100 µg i.v. dose of CPMV equates to 2.52 EU kg^−1^ LPS, which is below the FDA acceptable 5 EU kg^−1^ levels.^[^ ^51^ ^]^ The immunogenicity assay using RAW‐BLUE cells further confirmed that the low LPS levels did not contribute to the immune stimulation (Figure 6b).

The ability of the H6/G3 peptides to direct cargo to the TME was previously demonstrated when H6 and G3 peptides were conjugated to the Fc region of mouse IgG2b antibodies to specifically target S100A9 and deplete MDSCs within the TME.^[^ ^22^ ^]^ Previous work has also explored using small molecule drugs and neutralizing antibodies to block S100A9 function.^[^ ^8^, ^21^, ^22^ ^]^ However, to the best of our knowledge, S100A9 has never previously been targeted in immunotherapy. The exact functional role of S100A9 in cancer and tumorigenesis is not entirely understood, but the protein acts upon immune and tumor cells to modulate the TME into an immunosuppressive state thereby promoting tumor progression and aggressiveness.^[^ ^17^, ^52^, ^53^, ^54^, ^55^ ^]^ One hypothesis was that targeting S100A9 could block its function and delay tumor progression—however, S100A9‐targeted CCMV particles showed no efficacy even after lung homing (29–35% distribution). Considering the biodistribution data (Figure 3), it is suggested that the therapeutic effect is achieved exclusively by the unique potency of CPMV with the S100A9 serving solely as a molecular target.^[^ ^31^ ^]^ This was also validated by the RAW‐BLUE data (Figure 6a,b) in which RAW‐BLUE cells were only stimulated with CPMV and not with CCMV and the peptides.

Questions remain as to why the CPMV and CCMV nanoparticles home to the lungs of healthy mice (Figure 3). We hypothesize that endogenous levels of S100A9 within the lungs could be directing the nanoparticles to the lung microenvironment; S100A9 is a key mediator in fighting off pathogens within the lungs and instigating immune responses.^[^ ^56^ ^]^ In support of this point, it has been found that S100A9 becomes strongly upregulated by bronchial epithelial cells after LPS stimulation in vitro,^[^ ^57^ ^]^ is upregulated following tuberculosis and influenza A infection as well as other pathogens,^[^ ^58^, ^59^ ^]^ and improves the resistance of mucosal epithelial cells to bacterial invasion.^[^ ^56^ ^]^ S100A9 also comprises up to 45% of intracellular neutrophil proteins, but can be released into the extracellular space.^[^ ^9^ ^]^ A large part of the marginated neutrophil pool in healthy mice is found within the lungs, which may be one of the reasons for VNP biodistribution to the lungs.^[^ ^60^ ^]^

Especially encouraging was the fact that the S100A9‐targeted CPMV treatment demonstrated efficacy when used as a prophylactic and therapeutic immunotherapy (Figures 4 and 5). In the prophylaxis setting, CPMV‐H6 and CPMV‐G3 formulations were able to decrease tumor nodules by 14.8 (*p* < 0.0001) and 3.5‐fold (*p* = 0.0002) compared to PBS in the B16F10 murine melanoma model (Figure 4c). Histological examination of the lungs also demonstrated that CPMV‐H6 decreased the percentage of tumor cells by 18‐fold (*p* < 0.0001) while CPMV‐G3 decreased it by fivefold (*p* < 0.0001). Similarly, when tested against a murine TNBC model, CPMV‐G3 particles decreased tumor nodule counts by 99‐fold (*p* = 0.0005) and delayed tumor growth (Figure 4i, Figure S7b, Supporting Information). In both prophylactic studies, the CPMV particle without targeting showed some degrees of efficacy. In the melanoma and TNBC tumor models, CPMV decreased the number of tumor nodules by 2.1 (*p* = 0.02) and 10.6‐fold (*p* = 0.0015), respectively, compared to PBS. This is most likely attributed to the ability of the CPMV nanoparticle to induce systemic immune responses;^[^ ^26^, ^29^ ^]^ however, S100A9‐targeted CPMV outperformed native CPMV. For instance, in the B16F10 repeat study (Figure 4d), CPMV‐H6 decreased tumor nodule counts by 3.3‐fold (*p* = 0.077) compared to native CPMV. The improved efficacy of the S100A9‐targeted formulations can be attributed to successful tissue targeting of the lung resulting in the immunomodulation of the lung tissue microenvironment favorable for metastatic tumor cell rejection.

In the therapeutic studies, the targeted CPMV was similarly able to improve clinical outcomes in both the melanoma and breast cancer studies (Figure 5). CPMV‐H6 administration decreased B16F10 tumor nodules by 2.7‐fold compared to PBS (*p* < 0.0001) and 2.3‐fold compared to native CPMV (Figure 5b). In the 4T1‐Luc study, CPMV‐H6 slowed tumor growth and increased the median time of survival by 3 days (*p* = 0.0077). Contrary to the prophylaxis studies, native CPMV showed insignificant benefit. Average B16F10 tumor nodule count with CPMV was 1.2‐fold lower (*p* = 0.36) compared to PBS, and CPMV was unable to slow 4T1‐Luc tumor growth. This data indicates that systemically administered CPMV nanoparticles may have an ability to modulate immune‐mediated clearance of circulating tumor cells; however, after tumor cells establish in tissue, localized immune‐modulation is required.

The FACS data shed insight into the mechanisms behind the CPMV‐induced immunogenicity (Figure 6). Once the CPMV enters into the lung, it begins a cascade of events leading to stronger immune cell recruitment and activation (Figure 6c). Specifically, DCs, neutrophils, and macrophages were recruited to the lungs by administration of CPMV‐H6; DCs were increased by 40–50% (*p* < 0.0001), neutrophils by 70–80% (*p* < 0.0001), and macrophages by 50–70% (*p* < 0.0001) compared to controls. Surprisingly, CPMV‐G3 did not significantly improve immune cell recruitment; however, it did increase the number of active DCs by 12.1‐fold (*p* < 0.0001) and polarized 10.8‐fold (*p* < 0.0001) more M1 tumor‐killing macrophages compared to PBS. M1 macrophages are potent tumor cell killers and have tumor‐homing properties.^[^ ^61^ ^]^ You et al. have shown that the number of M1 populations within the tumor islets in non‐small cell lung cancer was positively correlated with patient survival.^[^ ^62^ ^]^ DC activation can reduce immunosuppressive DC states and decrease tumorigenesis through the priming of cytotoxic T‐cells and the release of immunostimulatory cytokines such as IL‐12, IFN‐*γ*, and Fms‐related tyrosine kinase 3.^[^ ^63^, ^64^ ^]^ Finally, the peptide‐conjugated CPMV particles targeted MDSCs better than the non‐targeted controls (Figure S6, Supporting Information). Others demonstrated that H6/G3 peptibodies target and deplete MDSCs.^[^ ^22^ ^]^ Whether CPMV‐H6/G3 targeting of MDSCs leads to blocking of the S100A9 axis is unknown, but it may be a contributing factor in addition to the immunostimulatory effect and therefore enhance efficacy. Cell migration and invasion assays may provide clues. However, the fact that CCMV‐H6/G3 did not confer any efficacy may indicate that simply targeting the MDSCs is not sufficient to achieve anti‐tumor effect in either the prophylactic or therapeutic setting. Rather it is the combination of targeting the lung microenvironment as mediated by the H6/G3 targeting ligands followed by the immunostimulatory nature of the CPMV adjuvant.

Together, data indicate that CPMV is a versatile cancer immunotherapy and its use could be extended beyond localized in situ treatments. As with other nanoparticle‐based therapeutics i.v. administered CPMV is cleared by the liver, therefore we also tested the hepatotoxicity by measuring serum alanine transaminase and aspartate transaminase (Figure S10, Supporting Information). After an initial increase in liver enzymes, physiological levels were restored within three days post treatment. We only examined liver toxicity as most of the VNPs are cleared within the liver (Figure 3). However, detailed CPMV organ toxicity has been previously examined at a dose 10x higher compared to dosage used in our studies, and no apparent toxicities were reported.^[^ ^35^ ^]^ In future studies more detailed immunotoxicity and pharmacology will be considered to pave the way for translational development. Further, combination therapies could be considered. We have already demonstrated that CPMV treatment synergizes with checkpoint blockade,^[^ ^65^ ^]^ chemotherapy,^[^ ^31^ ^]^ and radiation.^[^ ^25^, ^27^ ^]^ Finally, CPMV prime‐boost administration schedules could be established or slow‐release could be programmed through applications of long‐lasting formulations with microneedles, polymers, scaffolds, or metal‐organic frameworks.^[^ ^46^, ^66^, ^67^ ^]^

## 4 Conclusion

Metastatic tumors remain one of the most challenging sectors in oncology to both treat and diagnose. S100A9 has been recognized as a targetable protein with high expression in multiple tumor types. Here we demonstrate that S100A9‐targeted nanoparticles from CPMV home to the lungs. When administered prior to tumor challenge, S100A9‐targeted CPMV treatment allows local immunomodulation of innate immune cells and subsequent rejection of tumor cells in lung (prophylaxis). The same treatment of S100A9‐targeted CPMV was also effective when administered after lung metastatic tumor establishment (i.e., by significantly delaying tumor growth and improving overall survival rate). We envision this therapy to be a powerful prophylactic approach for high‐risk patients such as for those undergoing surgery from primary melanoma or breast cancer, where recurrence and outgrowth of metastatic disease are the main clinical challenges.

## 5 Experimental Section

### Materials and Cells

RPMI‐1640 medium, Hank's balanced salt solution, Dulbecco's modified Eagle's medium (DMEM), and PBS were purchased from Corning Life Sciences. Fetal bovine serum (FBS) was purchased from Atlanta Biologicals. Bovine serum albumin (BSA) was purchased from Sigma‐Aldrich. Penicillin/streptomycin, potassium phosphate monobasic and dibasic anhydrous powders, 4‐(2‐hydroxyethyl)‐1‐piperazineethanesulfonic acid (HEPES) buffer, sodium acetate anhydrous (NaOAc), methanol, glacial acetic acid, and Sulfo‐Cyanine5 (Cy5)‐NHS esters were purchased from Thermo Fisher Scientific. Dimethyl sulfoxide (DMSO), maleimide‐polyethylene glycol_8_‐succinimidyl ester (SM(PEG)_8_), sucrose, 10% (v/v) neutral‐buffered formalin solution, ethylenediaminetetraacetic acid (EDTA), and Bouin's solution were purchased from Sigma‐Aldrich. D‐luciferin potassium salt was purchased from Gold Biotechnologies. Ethanol (EtOH) was purchased from VWR International. Paraformaldehyde (PFA) was purchased from Electron Microscopy Sciences.

Mouse 4T1‐Luc (CRL‐2539‐LUC2) and B16F10 (CRL‐6475) cells were purchased from ATCC. 4T1‐Luc and B16F10 cells were passaged and grown in RPMI‐1640 and DMEM respectively and supplemented with 10% (v/v) FBS and 1% (v/v) penicillin/streptomycin. The cells were incubated at 37 °C in a 5% CO_2_ chamber. RAW‐BLUE Cells (Invivogen, San Diego, CA) were maintained in selection media containing Zeocin (Invivogen) and Normocin (Invivogen) as per instructions by the supplier.

### Preparation of Fluorescent‐Labeled and S100A9‐Targeted Cowpea Mosaic Virus and Cowpea Chlorotic Mosaic Virus

CPMV and CCMV nanoparticles were propagated in black eyed pea plants and purified as reported in previous work.^[^ ^34^, ^68^ ^]^ CPMV was kept at 10 mm potassium phosphate (KP) buffer (pH 7.0–7.2) to the concentration of 2 mg mL^−1^ while CCMV was kept in 10 mm NaOAc and 1 mm EDTA at pH 4.8 (from here on out called Buffer B).

SM(PEG)_8_ (5 equivalents) dissolved in DMSO was added to the CPMV particle solution and mixed at room temperature (RT) for 2 h. The solution was ultracentrifuged at 4 °C at 52 000 g for 1 h with a 40% sucrose cushion. The resulting pellet was resuspended in 10 mm KP, and 0.5 equivalents of H6 (MEWSLEKGYTIKGGGSC) or G3 (WGWSLSHGYQVKGGGSC) peptides were added and mixed at RT for 2 h.^[^ ^22^ ^]^ The solution was then dialyzed using a porous membrane tubing (12–14 kDa, Spectrum Labs) at RT overnight in 10 mm KP to remove unconjugated peptides.

CCMV nanoparticles were diluted to 2 mg mL^−1^ in 0.1 m HEPES buffer (pH 7.2). SM(PEG)_8_ (5 equivalents) was added and allowed to incubate at RT for 2 h. The buffer was exchanged to Buffer B using a 10 kDa molecular weight cut off (MWCO) filter, and the resuspended pellet was allowed to sit at RT for 2 h. The solution was ultracentrifuged at 52 000 g for 1 h, resuspended in buffer B, and diluted with 0.1 m HEPES. A half equivalent of the corresponding peptide (H6 or G3) was then added and mixed at RT for 2 h. The buffer was exchanged once more and pelleted with ultracentrifugation as before. The final pellet was resuspended in buffer B.

To prepare fluorescent CPMV, CPMV particles were diluted to 4 mg mL^−1^ and an equal number of equivalents of sulfo‐Cy5‐NHS esters and SM(PEG)_8_ were added. The particles were incubated for 2 h at RT shielded from light and centrifuged using a 100 kDa MWCO filter for 10–12 min at 14 000 g. The pellet was resuspended with 5 mm KP buffer and H6 and G3 peptides were added (0.5 equivalents) to the solution. The solution was mixed at RT for 2 h on an orbital shaker. The solution was then dialyzed using a 12–14 kD MWCO molecular porous membrane tubing (Spectrum Labs) at RT overnight in 10 mm KP.

CCMV fluorescent particles were diluted to 2 mg mL^−1^ in 0.1 m HEPES (pH 7.2) buffer. Equal number of equivalents of SM(PEG)_8_ and sulfo‐Cy5‐NHS esters were added, and the mixture was incubated at RT away from light for 2 h. The buffer was then exchanged to Buffer B using a 10 kDa MWCO filter and kept in buffer B for 2 h at RT before pelleting down through ultracentrifugation at 52 000 g for 1 h. The particles were resuspended in buffer B and diluted with 0.1 m HEPES before adding the H6 and G3 peptides (0.5 equivalents). The resulting solution was mixed at RT away from light for 2 h. The buffer was exchanged once more and pelleted with ultracentrifugation as before. The final pellet was resuspended in buffer B.

### SDS‐PAGE

CPMV and CCMV samples were diluted in 100 mm KP or 10 mm buffer B, respectively, and loaded with 4x lithium dodecyl sulfate Sample Buffer (Life Technologies) for a final concentration of 10 µg in 24 µL. The particles were then denatured at 95 °C for 5 min and loaded onto a 12% NuPAGE gel (ThermoFisher Scientific) and ran at 200 V, 120 mA, and 25 W for 40 min in 1x morpholinepropanesulfonic acid buffer (ThermoFisher Scientific). The gels were first destained in a mixture of deionized (DI) water, methanol, and acetic acid (50:40:10; v/v) for 30 min followed by staining in 0.25% (wt/vol) Coommassie Blue solution for 30 min before imaging with the AlphaImager system (Protein Simple).

### Agarose Gel Electrophoresis

CPMV particles were diluted in 100 mm KP; CCMV particles were diluted in 10 mm Buffer B. 6x Gel Loading Purple dye (Biolabs) was added to the CPMV samples. Instead of lithium dodecyl sulfate, glycerol (3 µL) was added to CCMV, and 5 µg of the virus particles were loaded onto a 0.8% (w/v; for CPMV) or 1% (w/v; for CCMV) agarose gel. With CCMV, the gels were run at 4 °C. The agarose gel was stained with 1 µL of GelRed nucleic acid gel stain (Gold Biotechnologies) and run for 30 min at 120 V and 400 mA. Immediately after the run, the gel was imaged using the AlphaImager system (Protein Simple) under UV light and then imaged again after staining with 0.25% (wt/vol) Coomassie Blue.

### DLS

A Zetasizer Nano ZSP/Zen5600 (Malvern Panalytical) was used for DLS measurements, and the CPMV and CCMV particles were diluted to 0.5 mg mL^−1^ in 10 mm KP and 10 mm buffer B, respectively. The particles were run at 25 °C with 3 measurements per sample. CPMV particles were also analyzed for surface charge using the Zetasizer Nano ZSP/Zen 5600. The particles were diluted to 0.3 mg mL^–1^ in 10 mm KP and run at 25 °C using the Smolvchowski method.

### FPLC

CPMV and CCMV were diluted to 0.1 mg mL^–1^ in 10 mm KP buffer or 10 mm Buffer B and run through a Superose 6 size‐exclusion column (column dimensions of 10 × 300 mm with an exclusion limit of 4 × 10^7^ M_r_) at 0.5 mL min^−1^ for a total volume of 50 mL in an ÄKTA Explorer FPLC machine (GE Healthcare LifeSciences). The elution profile was isocratic, and the UV detectors were fixed at 260 (nucleic acid) and 280 nm (protein).

### UV‐vis

UV–vis (Nanodrop 2000) was used to calculate the number of fluorescent dyes attached per particle as well as the concentration of VNPs in the solutions. The fluorescent CPMV and CCMV particles were diluted in 0.1 mm KP and 10 mm buffer B, respectively, and measured at 260, 280, and 647 nm to calculate the number of conjugated Cy5 particles per VNP. Concentration of the VNP solutions were carried out using the 260 nm wavelength readings. The extinction coefficients of CPMV, CCMV, and the Cy5 dye are 8.1 mL mg^−1^ cm^−1^, 5.85 mL mg^−1^ cm^−1^, and 270 000 cm^−1^ M^−1^, respectively.

### TEM

The CPMV and CCMV samples were imaged using a FEI Tecnai Spirit G2 BioTWIN TEM. The samples were loaded onto Formvar carbon film coated TEM supports with 400‐mesh hexagonal copper grids (VWR International) at concentrations ranging from 0.25 to 1 mg mL^−1^ in DI H_2_O for 2 min. The grids were washed with DI H_2_O twice for 45 s and then stained with 2% uranyl acetate (Agar Scientific) for 30 s twice. The samples were imaged at 300 kV.

### Biodistribution of CPMV and CCMV Virus Particles

All animals were purchased from The Jackson Laboratory and were housed at the Moores Cancer Center at the University of California, San Diego (UC San Diego). The animals were granted unlimited access to food and water, and all protocols and studies were compliant with the guidelines set out by the Institutional Animal Care and Use Committee of UC San Diego.

Healthy and B16F10 metastatic tumor‐bearing C57BL/6 female mice were used for biodistribution studies of the Cy5‐labeled CPMV and CCMV from Section 2. B16F10 cells (200 000 cells per mouse) were administered i.v. and the tumors were matured for one week. The mice were injected with PBS, CPMV‐Cy5, CPMV‐H6‐Cy5, CPMV‐G3‐Cy5, CCMV‐Cy5, CCMV‐H6‐Cy5, and CCMV‐G3‐Cy5 i.v. (*n* = 3, 200 µg). All samples were spun down at 11 200 g for 10 min and filtered through a 200 µm filter before this experiment and all future in vivo experiments to remove potential aggregates. After 24 h, the lungs were harvested and then imaged and quantified for fluorescence using the IVIS (Xenogen).

Further confocal imaging (Nikon A1R Confocal/TIRF STORM microscope) of B16F10‐inoculated mice lungs was accomplished using CPMV‐Cy5‐PEG and CPMV‐Cy5‐G3 particles (20 mg kg^−1^). After 6 h, mice were sacrificed and briefly perfused with 10 mL of PBS. The harvested lungs were embedded in optimal cutting temperature medium (Fisher Healthcare) and frozen using liquid nitrogen. They were sliced into 10 µm thick sections and mounted on microscope glass slides for immunofluorescence staining. OCT residue was removed using PBS. The tissue sections were blocked with 10% (w/v) BSA in PBS for 1 h and washed with PBS. Staining was accomplished with *α*‐S100A9 (1:100 dilution, R&D systems, AF2065) and fluorescently‐labeled secondary PE *α*‐goat IgG (1:20 dilution) antibodies prepared in 1% (v/v) BSA. The stained tissue samples were mounted on Fluoroshield with DAPI for confocal microscopy.

### MDSC Ex Vivo Targeting

Balb/c mice were inoculated s.c. with 5 × 10^4^ 4T1 cells per mouse. Cells from the tumor as well as splenocytes were harvested once the tumor volume reached 100 mm^3^. The tissues were digested by adding 60 µL of collagenase D (100 mg mL^−1^, ThermoFisher) and incubated for 1 h at 37 °C. After digestion, tissues were passed through 40 µm pore size strainers and centrifuged at 400 g for 5 min. The cell pellet was resuspended in 5 mL of 1x red blood cell lysis solution (eBioscience) and incubated at RT for 2 min, followed by the addition of PBS to stop red blood cell (RBC) lysis. Cells were centrifuged again at 400 g for 5 min and resuspended in PBS with 1% (w/v) BSA and 2 mm EDTA (FACS solution). Total cell count and viability were found using trypan blue solution (Sigma‐Aldrich) and a Countess automated cell counter (Invitrogen). Cells from tumor and splenocytes were adjusted to 3 × 10^6^ and 1 × 10^7^ cells mL^−1^, respectively, with FACS solution.

Immunofluorescence staining for flow cytometry was performed in a 96 well plate; 100 µL of cells were added to each well. Prior to incubation with VNPs, the cells were incubated with 0.5 µg of Fc block (CD16/32, BioLegend) per well for 10 min at 4 °C to block non‐specific binding.

Mouse CD45, Cd11b, Ly6G, and Ly6C markers were used for immunophenotyping for MDSC‐like cells in tumor and splenocytes. All flow antibodies were purchased from Biolegend and diluted in FACS solution according to the manufacturer's recommendation. The markers used were for monocytic MDSCs (CD11b, [M1/70]; Ly6G [1A8]; Ly6C [HK1.4]) and granulocytic MDSCs (CD11b, [M1/70]; Ly6G [1A8]; Ly6C [HK1.4]).

Three different staining solutions were prepared by mixing antibody solutions with the same number of each particle formulation (CPMV‐Cy5, CPMV‐Cy5‐H6, and CPMV‐Cy5‐G3). For staining, 200 µL of antibody solution with the particles was added to each well in a 96 well plate. Cells were incubated at RT for 1 h in the dark and then centrifuged at 500 g for 5 min. The supernatant was aspirated, and the plate was vortexed to loosen cells. Cells were washed by adding 300 µL of FACS solution and centrifugation was repeated. After staining, cells were fixed using 2% (w/v) PFA in PBS for 1 h, washed once, and resuspended in 150 µL FACS solution. The cells were stored at 4 °C overnight prior to measurement. BD LSRII (BD Bioscience) and FlowJo were used for data acquisition and analysis, respectively.

### B16F10 i.v. Challenge to CPMV and CCMV Pre‐Treated Mice (Prophylaxis)

C57BL/6 female mice were first treated by i.v. administration of 200 µg of CPMV, CPMV‐H6, CPMV‐G3, CCMV, CCMV‐G3, G3 peptide only, or PBS (*n* = 5). Total free peptide molecules was normalized based on the peptides displayed per CPMV (as determined by ImageJ analysis of separated coat proteins on SDS‐PAGE gels). After 7 days, the mice were challenged by i.v. administration of 200 000 B16F10 melanoma cells per mouse. Lungs were harvested at day 21 (day 14 after tumor inoculation) and fixed in a 10% (v/v) neutral‐buffered formalin solution overnight. Following fixation, the lungs were stored in 70% (v/v) ethanol (EtOH), and the number of tumor nodules per lung was manually counted.

After tumor nodule counting, the fixed lung samples were submitted to the La Jolla Institute for Immunology for H&E staining and imaging. Paraffin‐embedded blocks were sectioned at 4 mm on a Leica RM2125 RTS microtome. The sections were then floated on a 42 °C tissue flotation bath and mounted onto Fisher Superfrost Plus microscope slides and subjected to H&E staining. Scanning was accomplished using a ZEISS AxioScan Z1 using a 20x objective. The ratio of tumor cells to total cells was measured from the histology slides using QuPath software.^[^ ^69^ ^]^

### 4T1‐Luc i.v. Challenge to CPMV Pre‐Treated Mice (Prophylaxis)

Balb/c mice were first treated by i.v. administration of 225 µg CPMV‐G3, CPMV, or PBS (*n* = 4). After 5 days, the mice were challenged by i.v. injection of 200 000 4T1‐Luc cells. The mice were imaged with the IVIS using luminescent imaging every 3 days by injecting intraperitoneally (i.p.) 150 mg kg^−1^ of body weight D‐luciferin. ROI measurements were taken through the Living Image 3.0 software. The weight of the mice was also tracked every 3 days. After 25 days (20 days after tumor inoculation), the lungs were collected and fixed in Bouin's solution for 3 days. Each tumor nodule on the lungs was counted manually and averaged between the mice in each group.

### Treatment of B16F10 Melanoma and 4T1 Metastasis Using CPMV Particles

B16F10 cells (40 000 per mouse) were injected i.v. into female C57B6/J mice (*n* = 7–12). 3 days after tumor inoculation, the mice were injected i.v. with PBS, CPMV, CPMV‐H6, and H6 (200 µg per mouse). H6 peptide only controls were injected at 20% the number of total CPMV coat proteins as estimated from Image‐J analysis of peptide conjugation success. 15 days following particle injections, the organs were harvested and stored in 10% neutral buffered formalin solution overnight. The organs were moved to 70% (v/v) EtOH the next day and the tumor nodules were individually counted.

4T1‐Luc cells (100 000 per mouse) were injected i.v. into female Balb/c mice (*n* = 15). 3 days following tumor injection, the mice were split into three groups (*n* = 5) consisting of PBS, CPMV, and CPMV‐H6 treatments (200 µg per mouse). Tumor growth was monitored by total bioluminescence imaging based on the i.p. injection of 150 mg kg^−1^ of body weight D‐luciferin. Total bioluminescence was determined using the Living Image 3.0 software, and ROI were quantified as average counts.

### Immunogenicity Profile of CPMV and CCMV Particles In Vitro

The immunogenicity between CPMV and its peptide‐conjugated counterparts was assessed using a RAW‐BLUE assay (Invivogen). Briefly, 100 000 RAW‐BLUE cells per well were incubated with 10 µg of CPMV, CPMV‐H6, CPMV‐G3, LPS, and H6 and G3 peptide for 24 h. TLR and NOD stimulation was assessed by measuring the levels of secreted embryonic alkaline phosphatase using a QUANTI‐Blue (Invivogen) assay. Absorbance was measured at 655 nm using a Tecan microplate reader.

The immunogenicity of CPMV and CCMV was also compared through a RAW‐BLUE assay. 100 000 RAW‐BLUE cells per well were incubated with 0.5 µg of CPMV and CCMV, 1.023 EU mL^−1^ *E*. *coli* endotoxin standard control (ThermoScientific), or culture media for 18 h. The endotoxin standard control concentration tested was the calculated amount of LPS produced from purification of CPMV. A QUANTI‐Blue assay was run like before, and absorbance was measured at 655 nm.

### Flow Cytometry for Innate Immune Cell Profile In Vivo

C57BL/6 mice were treated i.v. using CPMV, CPMV‐H6, CPMV‐G3, or PBS (*n* = 3) at a dose of 200 µg per mouse. Lungs were harvested after 24 h. Harvested organs were each placed in separate gentle MACS C tubes (Miltenyi Biotec) and dissociated with enzymatic solutions (lung dissociation kits, Miltenyi Biotec). The lungs were digested and cells were collected as in Section 5, and cell concentrations were adjusted to 1.0 × 10^7^ cells mL^−1^.

The protocol from Section 5 was then followed without the addition of the VNPs except the following markers were used: DC (CD11b, [M1/70]; CD11c, [N418]), activated DCs (DC markers plus MHCII, [M5/114.15.2]; CD86, [GL‐1]); macrophages (CD11b, [M1/70]; Ly6G‐F4/80, [1A8]), M1 macrophages (macrophage markers plus MHCII, [M5/114.15.2]; CD86, [GL‐1]), and neutrophils (Ly6G, [1A8]; Ly6C, [HK1.4]; CD11b, [M1/70]).

### Aspartate Aminotransferase and Alanine Aminotransferase Liver Toxicity Assays

C57BL/6J mice (*n* = 5–7) were treated i.v. with CPMV, CPMV‐H6, CPMV‐G3, H6, G3, or PBS (200 mg CPMV per mouse; the peptide dose was normalized to match the number of peptides delivered by CPMV). After 1, 3, and 7 days, blood was collected through retroorbital bleeds using heparinized tubes (Fisher Scientific). The blood was spun down at 7500 rpm for 10 min at 4 °C and the sera was collected and stored at −80 °C. The sera were then subjected to aspartate aminotransferase (AST) and alanine aminotransferase (ALT) activity testing by following the manufacturer's guidelines (Abcam). Briefly, the sera were diluted 10x, and compared against a standard curve of pyruvate and glutamate for the ALT and AST assay, respectively. Fluorometric readings at 535 nm (excitation) and 587 nm (emission) and at 10 and 40 min were used to measure ALT activity while AST activity was measured using absorbance readings at 450 nm also at 10 and 40 min (Tecan plate reader).

### Statistical Analysis

All data points were analyzed directly without pre‐processing and are displayed as mean ± SD. The sample size (*n*) of the biodistribution and flow cytometry experiments were all *n* = 3. The tumor prophylaxis and treatment studies ranged from *n* = 4–12. Statistical significance was determined using either one or two‐way analysis of variance with significance defined as *p* < 0.05. Statistical significance from Kaplan‐Meier plots was analyzed using Mantel‐Cox tests and defined as significant when *p* < 0.05. All figures and data analysis were created andaccomplishedusing Prism5(GraphPadSoftware).

## Conflict of Interest

Dr. Nicole F. Steinmetz is a co‐founder of and has a financial interest in Mosaic ImmunoEngineering Inc. The other authors declare no conflict of interest.

## Author Contributions

Y.H.C., J.P., and H.C. contributed equally to this work. Y.H.C., J.P., and H.C. researched data for the article, made a substantial contribution to the discussion. N.F.S. and J.P. conceived the idea and N.F.S. oversaw the study and all activities. Y.H.C. and N.F.S. wrote the manuscript. All authors reviewed and edited the manuscript prior to submission.

## Supporting information

Supporting Information

## Data Availability

Data available on request from the authors.
