# Supplementary material for: S100A9‐Targeted Cowpea Mosaic Virus as a Prophylactic and Therapeutic Immunotherapy against Metastatic Breast Cancer and Melanoma
Source: Adv Sci (Weinh). 2021 Sep 14;8(21):2101796. doi: 10.1002/advs.202101796 (PMC8564454; doi:10.1002/advs.202101796)
Supplement: Supplementary file 1 — Supporting Information [file ADVS-8-2101796-s001.pdf]

© 2021 Wiley-VCH GmbH

ADVANCED SCIENCE
Open Access

Supporting Information

for *Adv. Sci.*, DOI: 10.1002/advs.202101796

S100A9-Targeted Cowpea Mosaic Virus as a Prophylactic and Therapeutic Immunotherapy Against Metastatic Breast Cancer and Melanoma

Young Hun Chung[1]†, Jooneon Park[2]†Δ, Hui Cai[2]†#, Nicole F. Steinmetz[1,2,3,4,5,6*]

Supplementary Information

Title: S100A9-Targeted Cowpea Mosaic Virus as a Prophylactic and Therapeutic Immunotherapy Against Metastatic Breast Cancer and Melanoma

Young Hun Chung[1]†, Jooneon Park[2]†Δ, Hui Cai[2]†#, Nicole F. Steinmetz [1,2,3,4,5,6*]

[1]Department of Bioengineering, University of California, San Diego, La Jolla, CA, United States
[2]Department of Nanoengineering, University of California, San Diego, La Jolla, CA, United States
[3]Department of Radiology, University of California, San Diego, La Jolla, CA, United States
[4]Institute for Materials Discovery and Design, University of California, San Diego, La Jolla, CA, United States
[5]Center for Nano-ImmunoEngineering, University of California, San Diego, La Jolla, CA, United States
[6]Moores Cancer Center, University of California, San Diego, La Jolla, CA, United States

† These authors contributed equally to the manuscript
Δ Present address: Fate Therapeutics, San Diego, CA 92121
# Present address: School of Pharmaceutical Sciences (Shenzhen), Sun Yat-Sen University, Guangzhou, 510275, China
*email Nicole F. Steinmetz: nsteinmetz@ucsd.edu

Table of Contents

1. Figure S1: CCMV bioconjugation strategy.

2. Figure S2: Characterization of CCMV, peptide-conjugated, and fluorescent CCMV particles.

3. Figure S3: Figure S3: Additional characterization of the non-fluorescent CPMV and CCMV particles.

4. Figure S4: Additional characterization of dual-tagged peptide and fluorescent CPMV particles.

5. Figure S5: Biodistribution of fluorescent CPMV and CCMV nanoparticles following administration.

6. Figure S6: MDSC *ex vivo* targeting.

7. Figure S7: 4T1-Luc immunoprophylaxis study.

8. Figure S8: S100A9-targeted CPMV immunotherapy against lung metastasis from i.v. injected 4T1-Luc breast cancer cells in mice.

9. Figure S9: The gating strategy used in Figure 6c of the flow cytometry analysis of CPMV particles ability to stimulate innate immune cells.

10. Figure S10: Liver enzyme assays to determine liver toxicity of the CPMV i.v. injections.

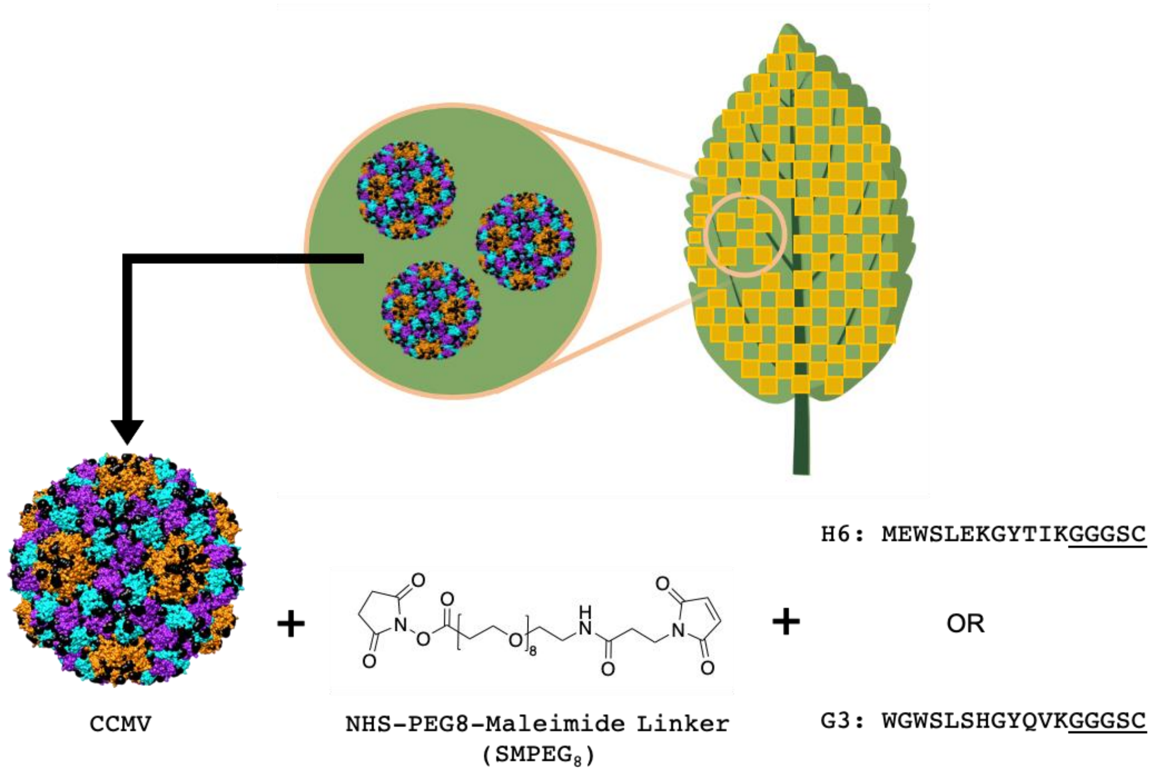

Figure S1: CCMV bioconjugation strategy. CCMV is first extracted from infected black-eyed pea No. 5 plants. Surface exposed lysines are highlighted as black spheres. The H6/G3 peptides with C-terminal Cys side chain (the linker is underlined) were then conjugated to CCMV using an $SMPEG_8$ linker *via* NHS-maleimide chemistry. CCMV images and chemical structures were drawn with UCSF Chimera and ChemDraw software. The image of the leaf was adapted from Biorender.com.

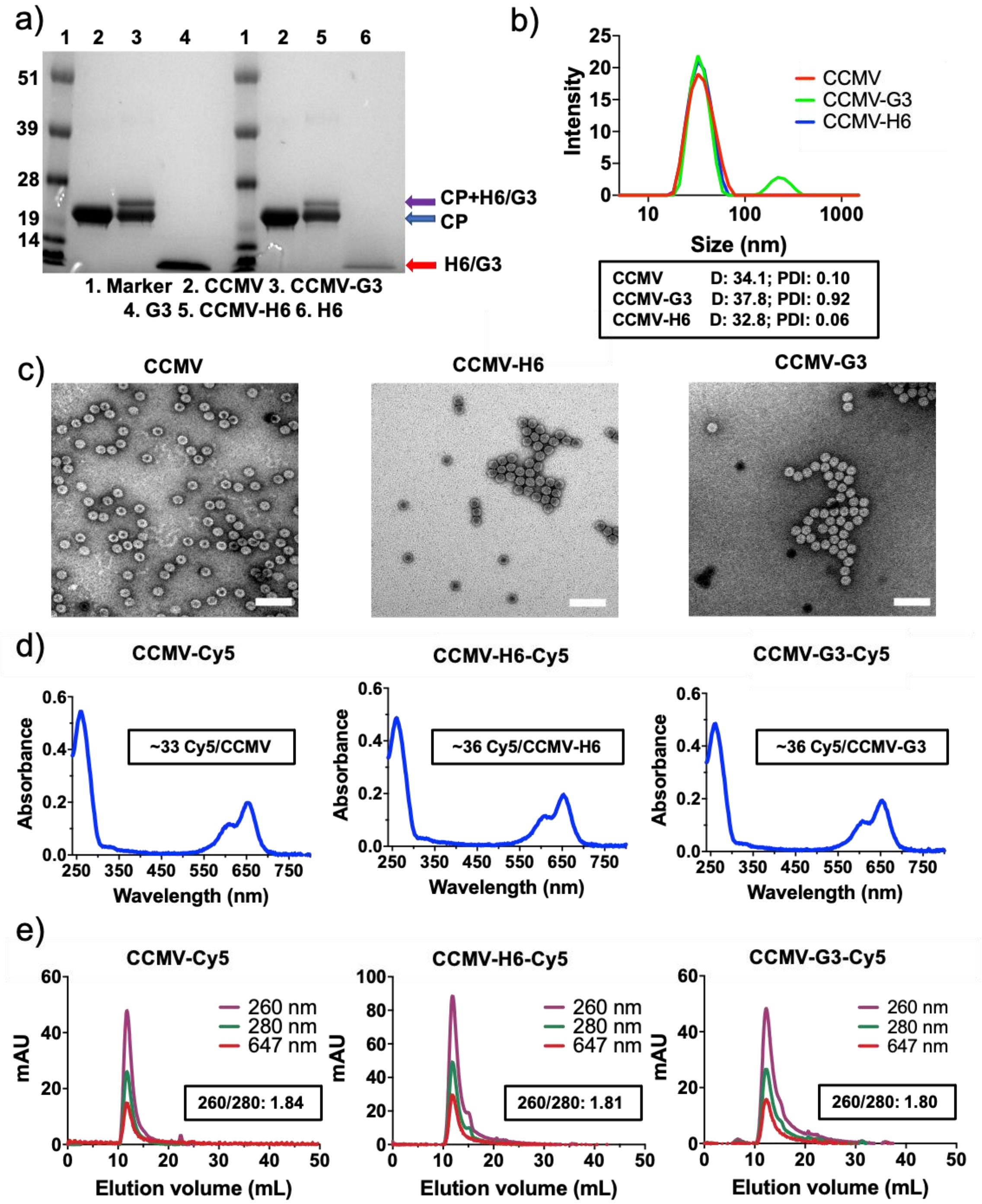

Figure S2: Characterization of CCMV, peptide-conjugated CCMV, and fluorescent CCMV particles. a) SDS-PAGE of the CCMV particles. The purple arrow points to H6/G3 peptide-modified coat proteins. The blue arrow points to the CCMV coat protein (21 kDa), and the red arrow points to the H6/G3 peptides. b) DLS measurements of the CCMV particles. The box in black is displaying the average diameter in nm of the particles (D) and the polydispersity index (PDI). The low PDI indicates minimal aggregation of the CCMV particles following bioconjugation. c) TEM images of uranyl acetate-stained CCMV particles. Scale bars represent 100 nm. d) UV-VIS of the fluorescent Cy5-conjugated CCMV particles. The boxed insets are displaying the number of conjugated Cy5 particles per CCMV particle. e) FPLC measurements of the dual fluorescent and peptide-conjugated CCMV particles. The inset is indicating the 260/280 nm ratio at the peak of the FPLC curve.

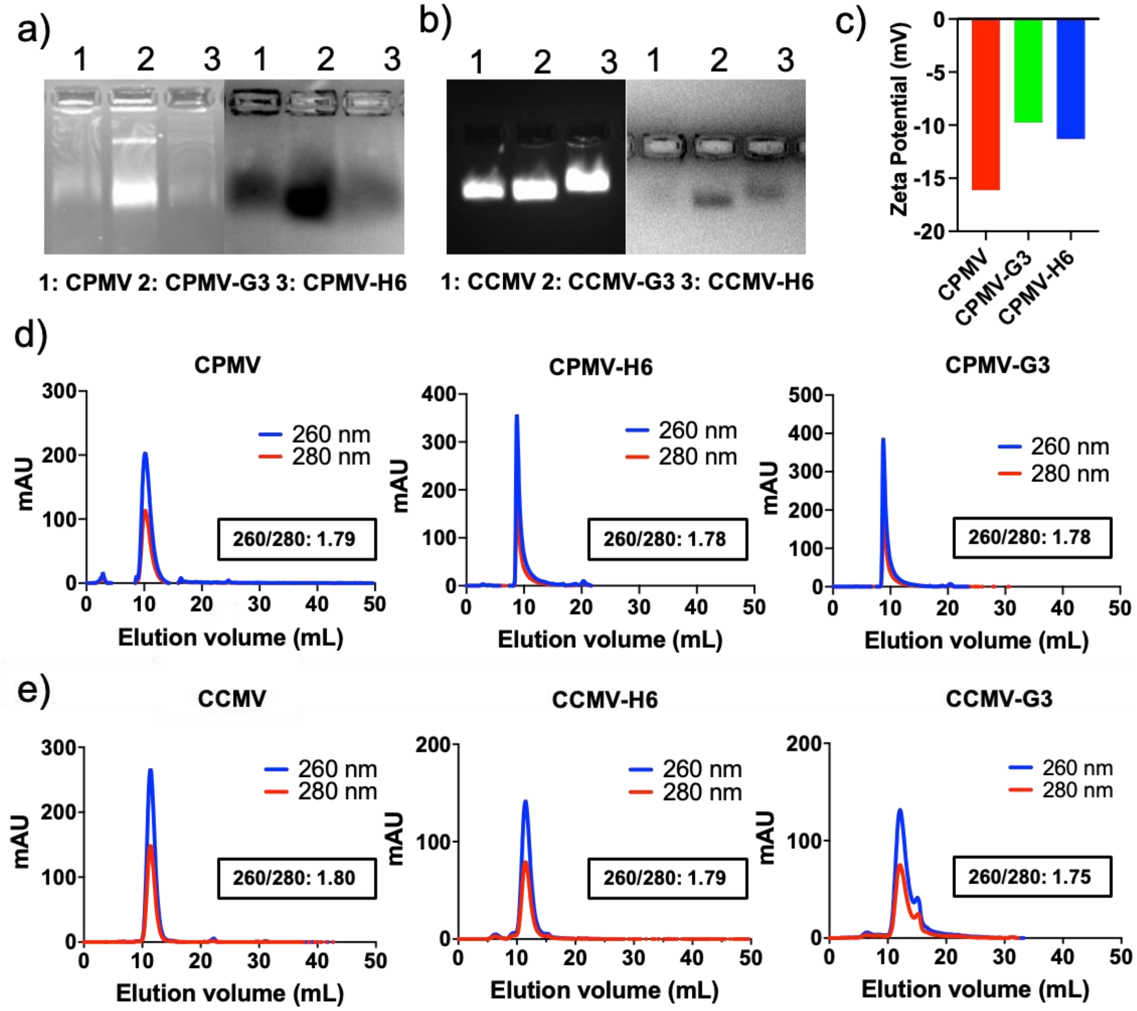

Figure S3: Additional characterization of the non-fluorescent CPMV and CCMV particles. a,b) 1.2% (w/v) agarose gel of the CPMV (a) and CCMV (b) particles. Data indicate that particles remain intact and do not aggregate considerably. c) Zeta potential analysis of the CPMV particles. The RNA agarose gel of CPMV has a lighter background compared to CCMV because the dye cannot penetrate the CPMV capsid as efficiently and images with CPMV have to be taken with higher intensities and longer exposure. d,e) FPLC graph of the CPMV (d) and CCMV (e) particles using a Superose 6 column and ÄTKA purifier. The inset is indicating the absorbance 260/280 nm ratio at the peak of the FPLC curve; an A260/280 ratio of 1.8 is indicative of intact particle preparations. The elution profiles are consistent with intact particles, although the left-shifted curve for CPMV may indicate some level of aggregation.

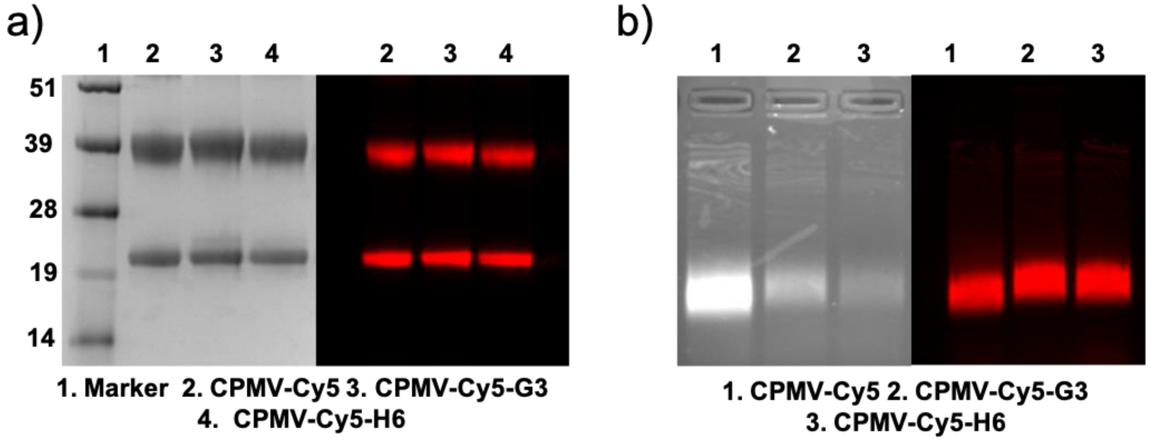

Figure S4: Additional characterization of dual-tagged peptide and fluorescent CPMV particles. a) SDS/PAGE gels of fluorescent CPMV particles. The Coomassie staining indicates successful conjugation to the peptides (as seen by the higher molecular weight bands) and the fluorescence (imaged at wavelength 647 nm) indicates that both the small and large coat proteins were successfully conjugated to the Cy5 fluorophore. b) 1.2% (w/v) agarose gels of the native and dual-tagged CPMV particles show that the particles remain intact. There is also minimal aggregation and no broken particles as indicated by the single bands.

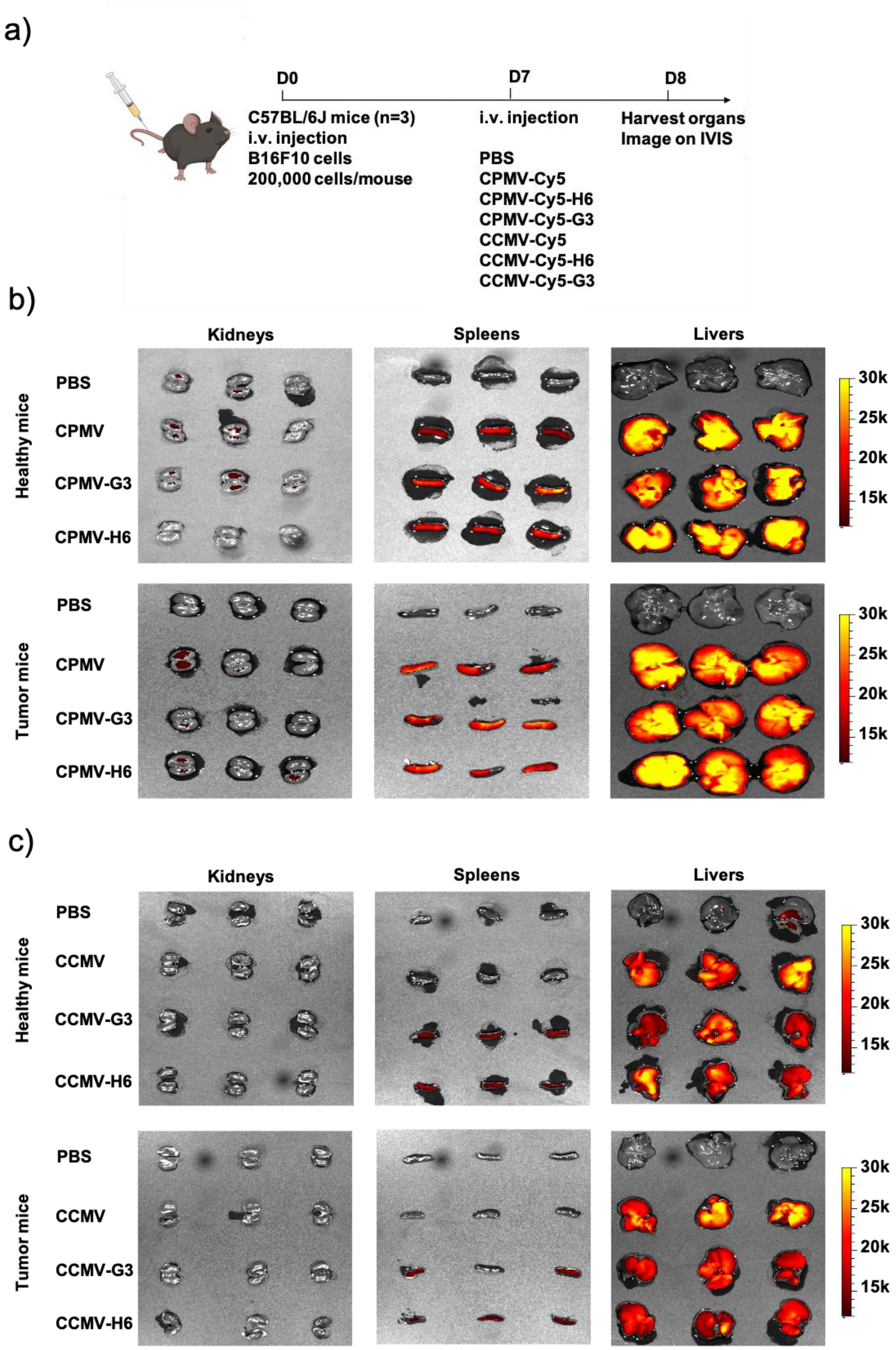

Figure S5: Biodistribution of fluorescent CPMV and CCMV nanoparticles following administration. a) Schematic and timeline of the biodistribution study. b,c) IVIS imaging of kidneys, spleens, and livers following CPMV (b) and CCMV (c) nanoparticle injection. All instances of CPMV and CCMV in Figures b,c are fluorescent nanoparticles, but were not labeled as Cy5-conjugated to improve image simplicity. The image of the mouse is created with BioRender.com.

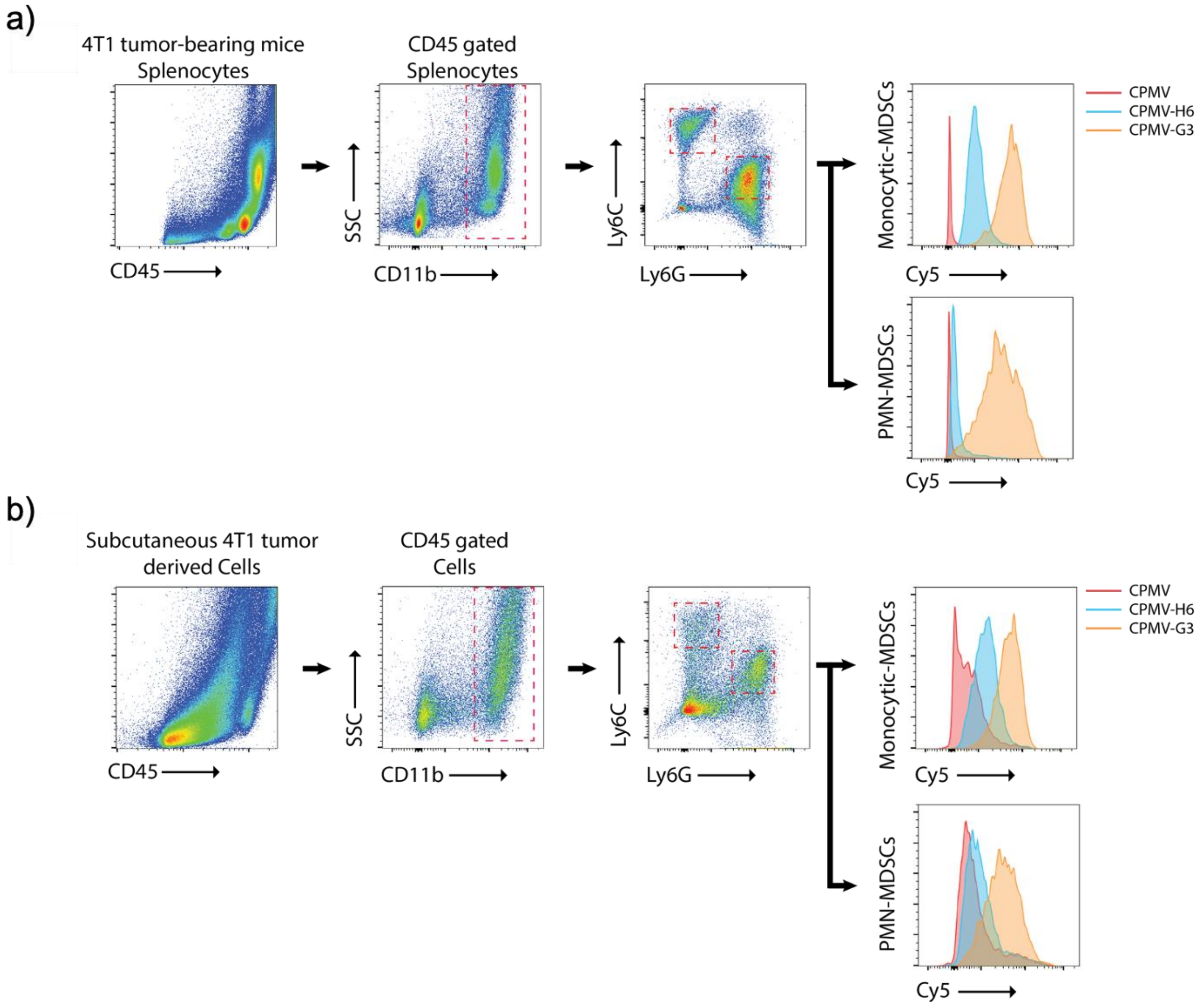

Figure S6: MDSC *ex vivo* targeting. Splenocytes (a) and tumor cells (b) from 4T1 tumor-bearing mice were harvested and investigated for CPMV, CPMV-H6, and CPMV-G3 binding using flow cytometry. Both monocytic and granulocytic MDSCs were targeted by the S100A9-specific CPMV-H6/G3 vs. native CPMV control with higher binding observed for CPMV-G3 vs. CPMV-H6, in particular in the PMN-MDSCs. PMN = polymorphonuclear, SSC = side scatter.

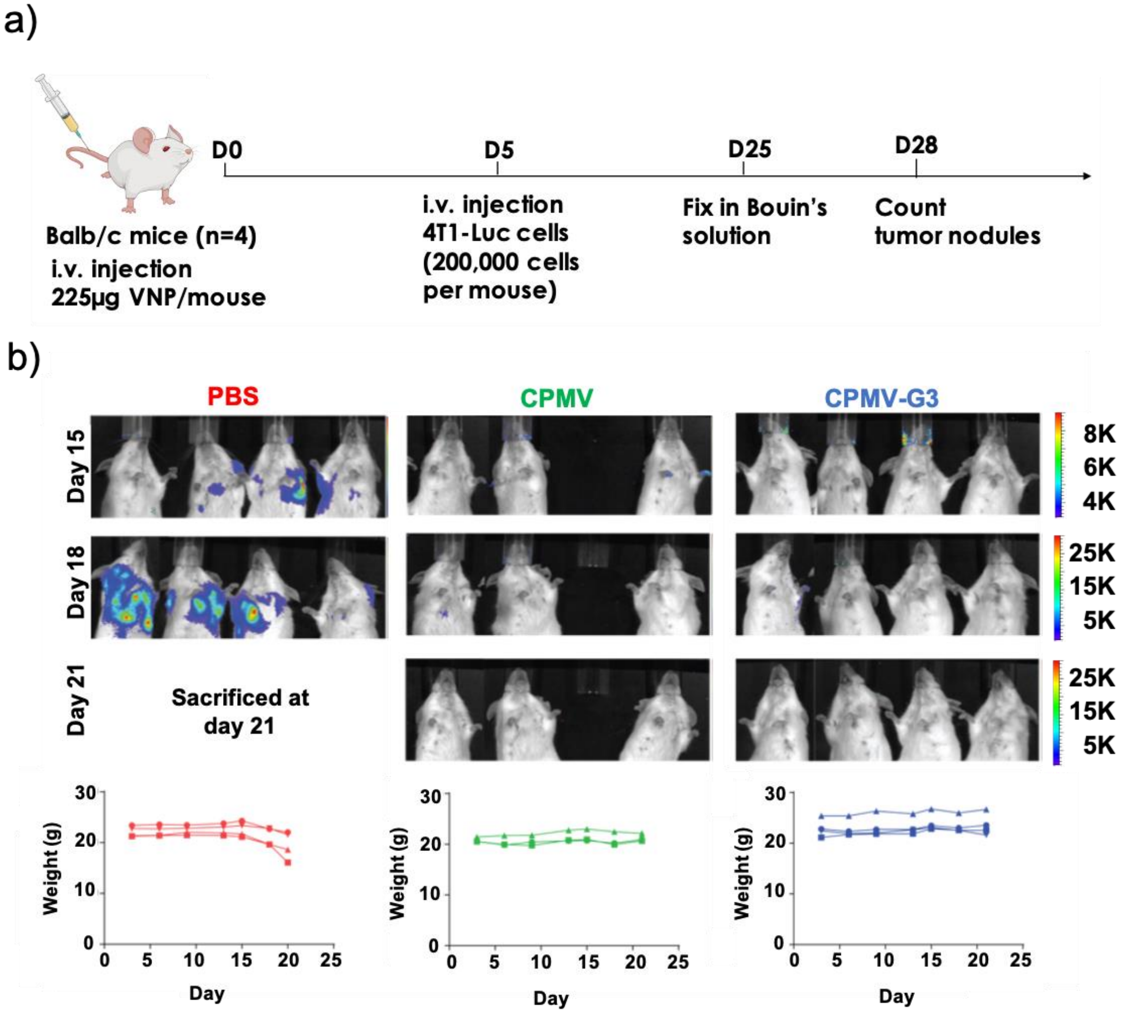

Figure S7: 4T1-Luc immunoprophylaxis study. a) Schematic and treatment schedule of CPMV as an immunoprophylaxis in 4T1-Luc metastatic breast cancer. b) Luminescent imaging of the 4T1-luc tumors taken every 3 days. D-luciferin was injected i.p. at a concentration of 150 mg/kg to enable luminescent imaging. Additionally, the mice were weighed and checked for any signs of noticeable weight loss. PBS mice were sacrificed by day 21 after meeting clinical endpoints and could not be imaged. The image of the mouse is created with BioRender.com.

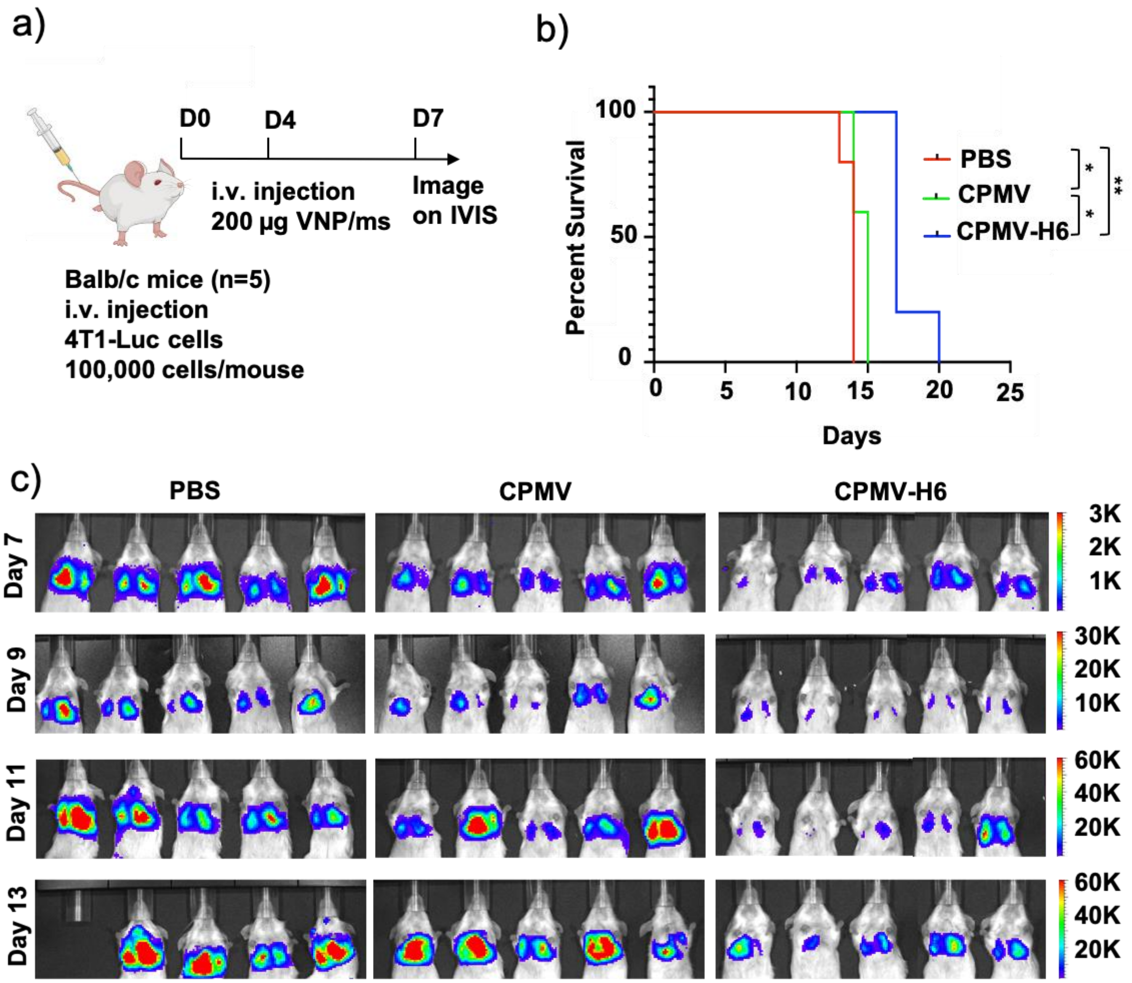

Figure S8: **S100A9-targeted CPMV immunotherapy against lung metastasis from i.v. injected 4T1-Luc breast cancer cells in mice.** a) Schematic and treatment schedule of the 4T1-Luc breast cancer model using Balb/c mice. b) Kaplan-Meier curve of the mice from the 4T1-Luc immunotherapy study. The mice were sacrificed when they reached their clinical endpoints. Wild type CPMV increased the median time of survival by one day while the CPMV-H6 treatment increased the median time of survival by 3 days. c) Luminescent imaging of the 4T1-Luc tumors taken on the IVIS. The mice were imaged every two days following 150 mg/kg i.p. injection of D-luciferin, and the luminescence was calculated using ROI measurements from the Living Image 3.0 software. Day 7 imaging was done at a lower scale, which may indicate why the tumor bioluminescence looks greater on Day 7 than Day 9. All experiments were accomplished with n = 5, and significance was deemed as $p < 0.05$. All analyses were done by one-way ANOVA. * = $p < 0.05$, ** = $p< 0.01$, *** = $p < 0.001$, **** = $p < 0.0001$, ns = not significant. The image of the mouse is created with BioRender.com.

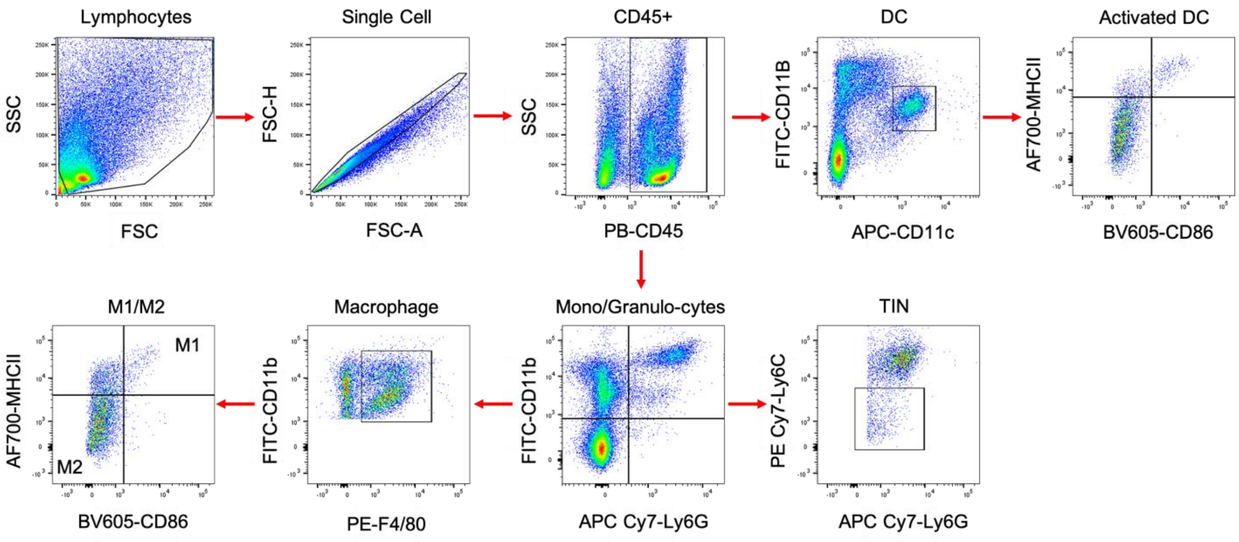

Figure S9: The gating strategy used in Figure 6c of the flow cytometry analysis of CPMV particles ability to stimulate innate immune cells. SSC = side scatter, FSC = forward scatter, DC = dendritic cell, TIN = tumor-infiltrating neutrophils

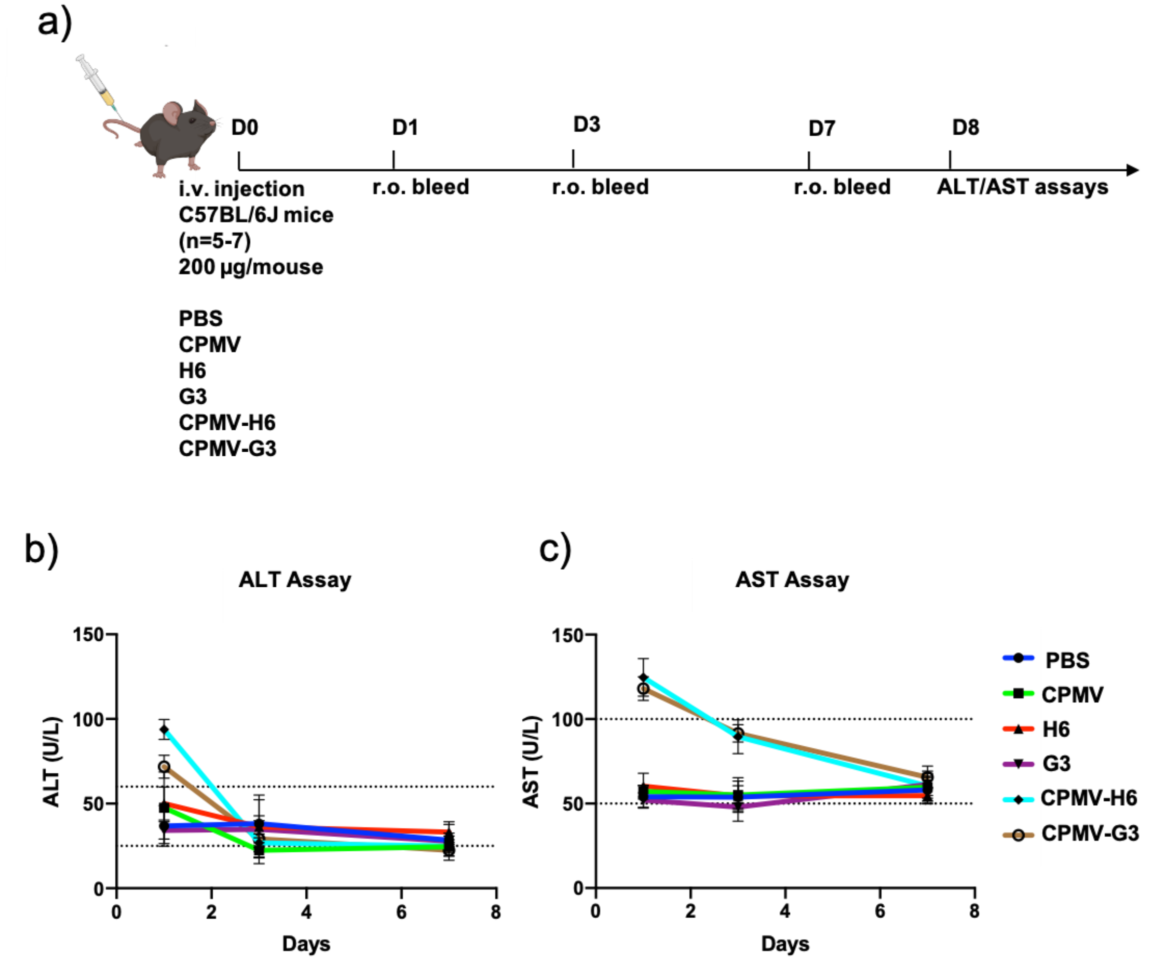

Figure S10: Liver enzyme assays to determine liver toxicity of the CPMV i.v. injections. a) Schematic of the ALT and AST assays. b) ALT Assay c) AST assay. The dotted lines in the ALT and AST assays represent the normal levels of ALT and AST in the blood of mice. The image of the mouse is created with BioRender.com.

Due to the strong accumulation of the CPMV particles in the liver (**Figure 3b,c**), we tested whether or not the CPMV particles were hepatotoxic in the mice. C57BL/6 mice (n = 5-7) were injected with 200 µg of CPMV, CPMV-H6, and CPMV-G3 as well as PBS, H6, and G3 controls (**Figure S10**). After 1, 3, and 7 days, the blood of the mice were collected and the sera was collected with centrifugation. The sera were then tested for both ALT and AST activity, which are both enzymes released in the blood following injection of hepatotoxic molecules.[1] The assays show that for both the ALT and AST assays, injection of CPMV-H6 and CPMV-G3 led to small increases in the ALT and AST systemic concentrations after 1 day (**Figure S10b,c**). However, by day 3 the enzyme levels were within the physiological ranges and the same was true for day 7. After significant liver damage, usually ALT and AST concentrations spike to 50 and 10-20x the normal levels[1] – our CPMV injections led to levels well below that range.

Works Cited

1. Huang, X.-J. *et al.* Aspartate Aminotransferase (AST/GOT) and Alanine Aminotransferase (ALT/GPT) Detection Techniques. *Sensors (Basel)* **6**, 756–782 (2006).
